# Preoperative assessment of liver regeneration using T1 mapping and the functional liver imaging score derived from Gd-EOB-DTPA-enhanced magnetic resonance for patient with hepatocellular carcinoma after hepatectomy

**DOI:** 10.3389/fimmu.2025.1516848

**Published:** 2025-01-30

**Authors:** Qian Li, Tong Zhang, Shan Yao, Feifei Gao, Lisha Nie, Hehan Tang, Bin Song, Yi Wei

**Affiliations:** ^1^ Department of Radiology, West China Hospital, Sichuan University, Chengdu, China; ^2^ MRI Research, GE Healthcare (China), Beijing, China; ^3^ Department of Radiology, Sanya People’s Hospital, Sanya, China

**Keywords:** liver regeneration, t1 mapping, gadolinium ethoxybenzyl DTPA, carcinoma, hepatocellular, hepatectomy

## Abstract

**Objectives:**

To explore whether T1 mapping parameters and the functional liver imaging score (FLIS) based on Gd-EOB-DTPA MRI could evaluate liver regeneration after hepatectomy for HCC patient.

**Methods:**

This retrospective study finally included 60 HCC patients (48 men and 12 women, with a median age of 53 years). T1 relaxation time of liver before gadoxetic acid injection (T1_pre_) and during the hepatobiliary phase (T1_HBP_), reduction rate (Δ%) and FLIS were calculated, their correlations with liver fibrosis stage, hepatic steatosis, and liver regeneration, quantified as regeneration index (RI), were assessed by Kendall’s tau-b correlation test or Spearman’s correlation test. Multivariate linear regression analyses were used to explore the indicator of RI.

**Results:**

T1_pre_, T1_HBP_, Δ%, and FLIS manifested significant correlation with fibrosis stage (r = 0.434, *P* =0.001; r = 0.546, *P* < 0.001; r = -0.356, *P* =0.005; r = -0.653, *P <*0.001, respectively). T1_pre_ showed significant correction with steatosis grade (r = 0.415, *P* =0.001). Fibrosis stage and steatosis grade were associated with RI (r = -0.436, *P*<0.001; r = -0.338, *P* =0.008). Accordingly, T1_pre_, T1_HBP_ and FLIS were the significant predictors (*P*<0.05) of RI in multivariate analysis. Similarly, in the patients undergoing minor hepatectomy (n=35), T1_HBP_, Δ% and FLIS were related to RI (*P*<0.05) in multivariate analysis. Nevertheless, in the patients undergoing major hepatectomy (n=25), no T1 mapping parameter and FLIS was the independent predictor of RI.

**Conclusions:**

T1 mapping parameters and FLIS were the potential noninvasive indicators of liver regeneration, except for HCC patients undergoing major hepatectomy.

**Clinical relevance statement:**

The value of T1 mapping and FLIS with Gd-EOB-DTPA MRI for accurate preoperative evaluation of liver regeneration is critical to prevent liver failure and improve prognosis of HCC patients.

## Highlights

- Accurate preoperative evaluation of liver regeneration is critical to prevent liver failure.
- T1 mapping parameters and FLIS were associated with fibrosis stage and liver regeneration.
- T1 mapping parameters and FLIS were the potential noninvasive indicators of liver regeneration for HCC patients.

## Introduction

Hepatocellular carcinoma (HCC) is the sixth most common malignant tumor and the third leading cause of cancer-related death worldwide (1). Although liver regeneration could occur after the first-line curative treatment for HCC, i.e., surgical resection, liver impairment or injury may exceed its hyperplastic ability, leading to post-operative liver failure (2, 3). Hence, accurate preoperative evaluation of liver regeneration (LR) is critical to the prognosis assessment and clinical management.

Advanced liver fibrosis and fatty liver are closely associated with the poor capacity of LR (4, 5). Currently, liver biopsy, magnetic resonance elastography (MRE), shear wave elastography (SWE), Intravoxel incoherent motion (IVIM) diffusion-weighted imaging and texture analysis have been used for preoperative evaluation of liver fibrosis and hepatic steatosis. However, invasiveness, sampling error, interobserver variability, low stability, the need of specific equipment and field strength dependency limit their clinical application (6–10). Evidence has proved that the change in the cell function and histological characteristics, concentration of extracellular matrix proteins, and activation of hepatic stellate cells, accompanied with the progression of liver fibrosis, would result in the changes in T1 relaxation time in fibrotic tissues (11, 12). Besides, hepatic steatosis induces a mixture of water and fat, and the fat is generally out-of-phase with water. In out-of-phase mixture of water and fat, the signal is subtracted by the fat component, resulting in slow T1 recovery and longer T1 relaxation time (13, 14). Liver fibrosis could also alter the expression levels of organic anion transporting polypeptides (Oatps) and multidrug resistance associated protein (Mrp), leading to an increased T1 relaxation time of the liver parenchyma in hepatobiliary phase with Gd-EOB-DTPA-enhanced MR (15). The T1 mapping sequence based on Gd-EOB-DTPA-enhanced MR do not depend on the device itself and directly reflect the true T1 value of tissues, thus providing more reliable and less subjective quantitative evaluation of liver function (15). Previous studies (16, 17) have proven that these metrics derived from T1mapping of pre-contrast (T1_pre_) and 20-min hepatobiliary phase (T1_HBP_ and T1 reduction rate) were suitable for detecting liver fibrosis(≥ F2)(T1_pre_: AUC, 0.70-0.89; T1_HBP:_ AUC,0.86; T1 reduction rate;AUC,0.89), and these metrics were also significantly correlated with hepatic steatosis(T1_pre_: r=-0.695, *P*<0.001; T1_HBP_: r=0.263, *P*<0.046 and T1 reduction rate: r=-0.310, *P*=0.018). Meanwhile, a functional liver imaging score (FLIS) that takes into account three features of gadoxetic acid–enhanced MRI of the liver: enhancement quality, rate of biliary contrast excretion, and persistence of signal intensity in the portal vein, was also associated with the severity of diffuse liver disease, FLIS ≤ 3 and FLIS≥ 5 were the optimal cutoff for distinguish ALBI grade 3 (AUC, 0.974-0.994) and Child-Pugh A or diffuse liver disease (AUC, 0.93),respectively (18, 19). Hence, T1 mapping of pre-contrast, 20-min hepatobiliary phase and FLIS are essential for preoperative assessment of LR capacity.

According to the previous studies (7, 20, 21), the resected volume is of vital importance for LR, and the large resected volume with major hepatectomy may cause high regeneration indices, such as regeneration index (RI), and hide the importance of liver itself on liver regeneration. Thus, it is warrant to separate out the analysis for patients who had minor vs. major hepatectomies.

The aim of our study is to evaluate the capacity of LR by Gd-EOB-DTPA-enhanced T1 mapping and FLIS score in HCC patients, and go on subgroup analysis with the type of hepatectomy.

## Materials and methods

### Participants and data collection

**Figure 1:**
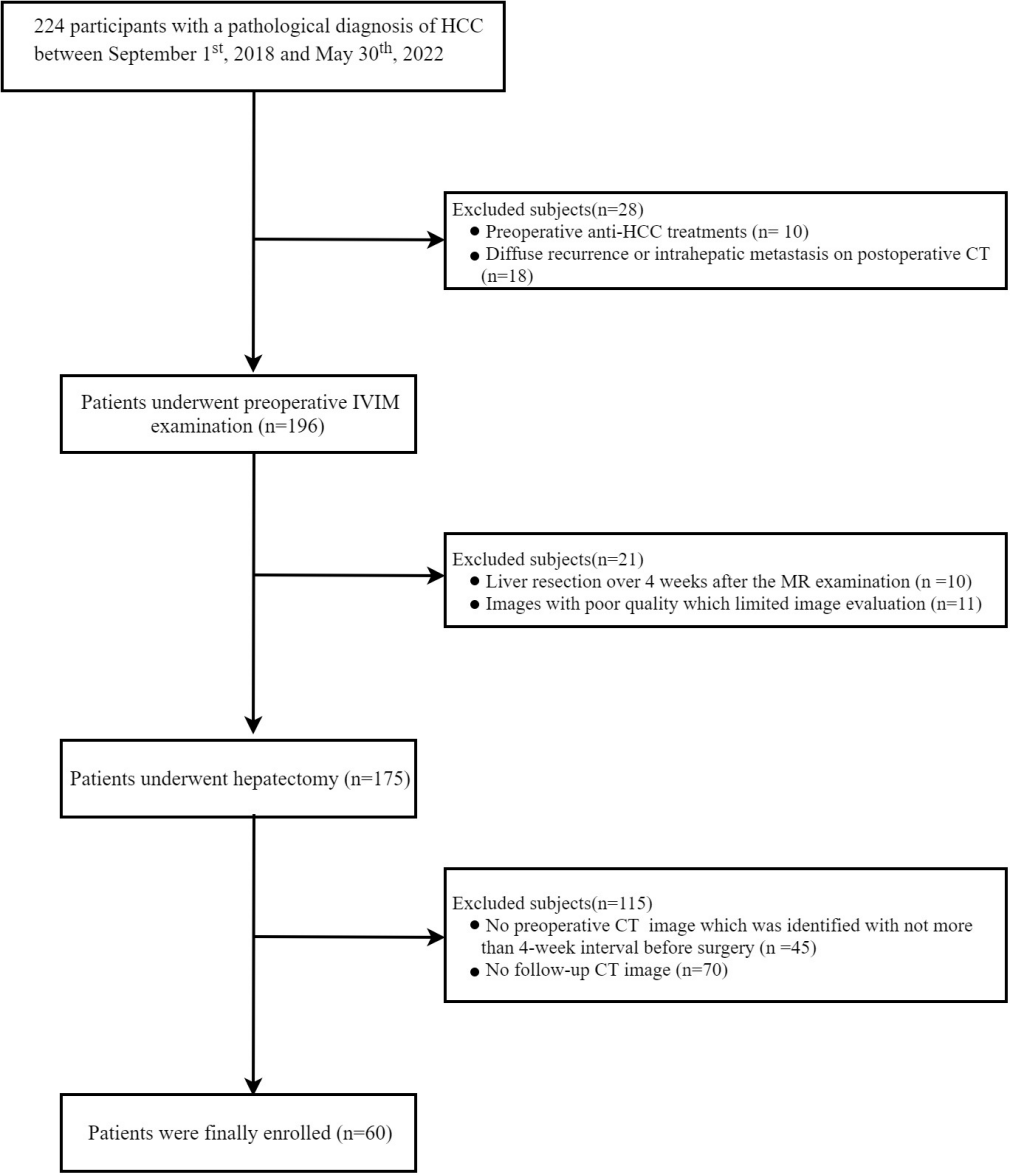
Flow diagram of the study population. RI, regeneration index.

This retrospective study was conducted in accordance with the ethical guidelines of the Declaration of Helsinki and was approved by the Institutional Review Board of Sichuan university, West China Hospital. Due to the retrospective nature, written informed consent was waived. A total of 224 pathological confirmed HCC patients, undergoing curative-intent hepatectomy and preoperative T1-mapping imaging examination derived from Gd-EOB-DTPA-enhanced magnetic resonance between September 2018 and May 2022, were consecutively recruited. The inclusion criteria were as follows: (1) patients were aged ≥18 years; (2) the time interval between preoperative T1-mapping imaging examination and hepatectomy was less than 4 weeks; (3) primary HCC without treatment; (4) have follow-up contrast-enhanced CT. Additionally, patients were excluded according to the following exclusion criteria (**Figure 1**): 1) received any anti-HCC treatments, such as hepatectomy, TACE, chemotherapy or radiofrequency ablation prior to hepatectomy, due to that anti-HCC treatment would cause uncertain impact on image quality, such as like signal interference and artifact, finally affecting the accuracy of imaging evaluation in liver parenchymal function. (n=10); 2) had diffuse recurrence or intrahepatic metastasis on follow-up computed tomography (CT) which would cause technical segmentation failure(n=18). The main reason was that the recurrent lesions and metastases spread throughout the liver parenchyma in patients with diffuse recurrence or intrahepatic metastasis, and it was difficult to segment hepatic parenchyma technically; 3) the time interval between hepatectomy and MR imaging was more than 4 weeks (n =11); 4) images with poor quality (e.g., severe artifact) that limited image evaluation (n=10); 5) no preoperative CT image from less than 4-weeks before hepatectomy (n=45); and 6) lacked follow-up contrast-enhanced CT in one year after hepatectomy (n=70). Finally, 60 patients with HCC were included in the final study cohort. Clinicopathologic variables, fibrosis stage confirmed by histological examination of a resected specimen (according to the METAVIR scoring system (22), F0-1 as no to mild fibrosis, F2 as significant fibrosis, F3 as advanced fibrosis, and F4 as cirrhosis), inflammation grade confirmed by histological examination of a resected specimen [according to the Scheuer scoring system (23)], steatosis grade confirmed by histological examination of a resected specimen [according to the amount of surface area of the parenchyma that was visually determined to be affected by steatosis (24), hepatic steatosis was divided into none (S0, < 5%), mild (S1, 5–33%), and moderate–severe (S2–3, > 33%)].

### MR imaging

A 3.0-T MR system (Discovery MR 750w, GE Healthcare) with a 16-channel phased-array torso coil (GE Medical System) was used for MRI examinations. Every patient fast for 6-8 hours before MR imaging. Saturation method using adaptive recovery times for T1 mapping sequence (SMART1Map) was used for T1 mapping scan. The parameters were as follows: TE/TR/flip-angle: 1.64msec/3.3msec/50°, slice thickness 8.0 mm, slice gap 2 mm, FOV 40 × 36 cm^2^, matrix 192 × 128, NEX 1. T1 mapping was performed before and 20 min after injection of Gd-EOB-DTPA (0.025mmol/kg; Primovist; Bayer Pharma AG, Berlin, Germany), which was administered intravenously using a bolus injection at the injection rate of 1 mL/s and followed by a 20-mL saline flush. T1 mapping images were then transferred to a T1 mapping and quantitative dynamic contrast enhanced MRI software package (Omni-Kinetics, GE Healthcare). And Omni-Kinetics using the VFA method automatically generated T1 maps.

### CT techniques

All patients underwent two multi-slice CT scan (preoperative CT and postoperative CT) by Revolution CT (GE Healthcare) or SOMATOM definition (Siemens). The scanning parameters were as follows: tube voltage: 100 kVp or 120 kVp, tube current: 200–450 mA, slice thickness: 1.5-5 mm, pitch: 0.992: 1, rotation speed: 0.5 s/rot, ASIR-V: 20%. All patients received an intravenous, nonionic contrast medium (iodine concentration, 300-370 mg/mL; volume, 1.5–2.0 ml/kg; contrast type, Iopromide Injection, Bayer Pharma AG) at a rate of 2-3 ml/s, and then a 20 mL saline was injected for the flush. The arterial phase and portal venous phases started at about 30-35 s and 60-75 s, respectively, after the contrast injection.

### Imaging analysis

Two independent radiologists (LQ and YW), with 10 and 6 years of experience in abdominal imaging, blinded to the clinical data, laboratory tests, and histopathological results, reviewed all the T1 mapping images. T1 mapping was generated automatically inline based on a pixel-by-pixel fitting. The ROIs were kept in the same position before and after enhancement. According to the resection margin based on postoperative contrast-enhanced CT image, the section of largest future remnant liver parenchyma was selected to measure related relaxation values by referring to the HBP imaging, avoiding visible vessels and artifacts and maintaining a 0.5 cm distance to the surface of the liver. The mean T1 relaxation time for three ROIs was considered as the representative T1 relaxation time for the liver. Δ% refers to the reduction rate of T1 relaxation time, which was calculated as [(T1_pre_) – (T1_HBP_)]/(T1_HBP_) × 100%, where T1_pre_ and T1_HBP_ are the T1 relaxation time of the liver before and 20 min after gadoxetic acid injection. The mean value measured by the two radiologists was used for further statistical analysis. Additionally, the respective measures obtained by the two radiologists were used to determine inter-observer agreement expressed in terms of the intra-class correlation coefficient (ICC).

Two independent radiologists (SY and TZ), with 6 and 12 years of experience in abdominal imaging, blinded to the clinical data, laboratory tests, and histopathological results, reviewed all HBP–enhanced images. The radiologists assigned an FLIS to each patient independently. When the FLIS ranged from 0 to 6 points and was calculated by summing scores for liver parenchymal enhancement, biliary contrast excretion, and portal vein sign (18, 25):

1. Enhancement quality score of 0, 1, or 2 compared the liver to right kidney uptake. A score of 0, 1, or 2 meant the liver was hypo-, iso-, or hyperintense, respectively, to the right kidney.
2. Excretion quality score of 0, 1, or 2 was determined on the basis of the degree of contrast agent excretion into the biliary tract. A score of 0, 1, or 2 meant there was no biliary tract contrast excretion, excretion into peripheral intrahepatic bile ducts or the right and/or left hepatic duct(s), or excretion into the common hepatic duct, the common bile duct, or the duodenum, respectively.
3. The portal vein sign quality score of 0, 1, or 2 was on the basis of the portal vein relative to liver parenchymal signal intensity. A score of 0, 1, or 2 meant the portal vein was hyper-, iso-, or hypointense to the liver parenchyma, respectively.

### Preoperative CT liver volume

The liver evaluation software embedded on a post-processing workstation (uWs-CT, R005, United-Imaging Healthcare, Shanghai, China) was used for liver volume assessment based on preoperative contrast-enhanced CT. The entire liver parenchyma and vessels (mainly including the hepatic artery, portal vein, hepatic vein, and their main branches) were automatically extracted by this software. Manual corrections of liver contours were performed by an experienced radiologist (TZ) with 10 years of abdominal CT experience when necessary. Secondly, a straight line along the maximum diameter of the tumor was manually drawn by the above experienced radiologist, and the tumor was then semiautomatically segmented. The volume of the total functional liver (removing tumor volume and vessel volume) was automatically calculated and displayed. Besides, the above experienced radiologist drew a virtual curve along the surgical margin according to the postoperative CT image. Finally, the volume of the preoperative remnant liver (LV_pre_ removing volume of vessels) was calculated automatically. The parenchymal hepatic resection rate (PHRR) (20) was calculated using the following equation:


$$\text{PHRR}=\frac{\left(\text{Volume}\;\text{of}\;\text{the}\;\text{total}\;\text{functionalliver}\right)-\text{LVpre}}{\text{Volume}\;\text{of}\;\text{total}\;\text{functional}\;\text{liver}}\times 100\text{\% }$$

### Postoperative CT liver volume

Since the retrospective nature and there was no routine protocol for postoperative follow-up CT, the timing of follow-up CT was varied. The remnant liver volume often regenerates dramatically in the 6th month after hepatectomy. Accordingly, the follow-up portal phase CT images acquired closest to the 6th month after surgery were utilized to calculate the volume of the postoperative remnant liver (LV_post_). As described previously, LV_post_ and major intrahepatic vessels were automatically extracted and manually corrected, and then the LV_post_ (after subtracting the volume of vessels) was automatically calculated. The regeneration index (RI) was calculated using the following equation:


$$\text{RI}=\frac{\text{LVpost}-\text{LVpre}}{\text{LVpre}}\times \;100\%$$

### Statistical analysis

Categorical variables were summarized as frequencies and proportions, while continuous variables were expressed as means with standard deviations or medians with interquartile ranges, depending on the distribution assessed by the Kolmogorov–Smirnov test. Independent sample t test or Mann–Whitney U test was used to assess the differences of baseline characteristics between patients undergoing minor or major hepatectomy for continuous variables, the chi-square test or Fisher’s exact test or Wilcoxon rank sum test was used for categorical variables. Correlations between baseline characteristics and RI were evaluated by Spearman’s correlation test. The relation between T1 mapping parameters and fibrosis stage, fibrosis stage and RI were assessed using Kendall’s tau-b correlation test. Multivariate linear regression analyses were used to find the factors related to RI, and only those parameters which had statistical significance (*P*<0.05*)* in Spearman’s correlation test were subsequently included in further multivariate regression analysis. VIF (Variance Inflation Factor) was used to evaluate multicollinearity between variables in multivariate analysis, and VIF >10 considered indicative of multicollinearity.

Intra-class correlation coefficient (ICC) with the two-way random method was used to check the interobserver agreement toward the diffusion parameters (values < 0.50 poor agreement, 0.51–0.75 moderate agreement, 0.76–0.90 good agreement, > 0.91 excellent agreement). PASS software was used to ensure robust sample size determination and power analysis in multiple linear regression. All statistical tests were performed using SPSS software (Version 26, IBM) and R software (Version 4.0.2). All *P* value less than 0.05 were considered significant.

## Results

### The baseline characteristics of the included patients

Finally, a total of 60 HCC patients (48 men and 12 women, with a median age of 53 years) were included in this study, and the sample size was in the adequate range for the study. The follow-up period from surgery to postoperative CT scans ranged from 1.5–11.5 months. LV_pre_, LV_post_, PHRR and RI of all the patients were 729.18 ± 32.25, mL; 1022.29 ± 28.83, mL; 38.61 ± 2.40, %;50.84 ± 6.08, %, respectively. There were 25 patients receiving major hepatectomy, and the remaining patients (n=35) underwent minor hepatectomy. Compared with the patients undergoing major hepatectomy, these undergoing minor hepatectomy had higher LV_pre_ (867.50 ± 201.31, mL VS. 535.54 ± 170.40.52, mL, *P*<0.001) and LV_post_ (1081.58 ± 197.61, mL VS. 939.28 ± 234.42, mL, *P*=0.014), lower PHRR (26.57 ± 12.35, % VS. 55.47 ± 11.22, %, *P*<0.001) and RI (27.91 ± 24.82, % VS. 82.94 ± 52.39, %, *P*<0.001). However, the other clinical characteristics were not significant different (all *P*>0.05)between the two subgroups. Detailed information about the baseline characteristics were summarized in **Table 1**.

**Table 1:** Baseline characteristics of the included patients.

Baseline characteristics	Total patients (n=60)	Minor hepatectomy (n=35)	Major hepatectomy (n=25)	*P* value
Clinical characteristics				
Age (years)	53.00 (44.00,58.00)	55.50 (43.75,60.25)	51.00 (42.50,56.00)	0.103
Gender				0.513
Male	48 (80.00)	27 (77.14)	21 (84.00)	
Female	12 (20.00)	8 (22.86)	4 (16.00)	
BMI	23.78±0.45	23.73±4.04	23.67±2.51	0.946
ALT (IU/L)	37.00 (23.00,63.50)	33.00 (21.75,48.50)	44.00 (28.50,68.00)	0.081
AST (IU/L)	35.50 (25.50,51.00)	28.50 (22.50,38.00)	36.00 (28.60,42.00)	0.082
ALP (IU/L)	91.00 (80.00,136.25)	88.00 (76.75,118.25)	83.50 (73.00,96.00)	0.254
GGT (IU/L)	71.00 (38.50,146.25)	48.00 (30.75,85.75)	60.00 (41.20,92.00)	0.006
ALB (g/L)	42.45±0.70	41.94±5.46	43.16±5.39	0.397
TBIL (umol/L)	13.60 (10.55,16.63)	12.55 (9.55,16.40)	14.80 (11.60,19.05)	0.146
DBIL (umol/l)	4.85 (2.40,6.00)	4.10 (3.28,5.43)	5.20 (4.00,7.20)	0.057
HGB (g/L)	141.76±2.68	140.38±20.91	143.04±19.91	0.624
PLT (10^9/L)	157.90±10.34	151.88±76.40	168.48±81.76	0.427
PT (s)	11.35 (10.90,11.93)	11.10 (10.70,11.70)	11.60 (11.25,12.35)	0.684
INR	1.01 (0.96,1.05)	1.00 (0.96,1.05)	1.02 (0.98,1.08)	0.113
HbsAg (Positive, %)	51 (85)	29 (82.86)	22 (88)	0.722
HBeAg (Positive, %)	5 (8.33)	5 (14.29)	0 (0)	0.069
Anti-HCV (Positive, %)	0 (0)	0 (0)	0 (0)	1.000
ALBI grade				0.337
I	48 (80.00)	27 (77.14)	21 (84.00)	
II	10 (16.67)	6 (17.14)	4 (16.00)	
III	2 (3.33)	2 (5.71)	0 (0.00)	
LVpre (mL)	729.18±32.25	867.50±201.31	535.54±170.40	<0.001^*^
LVpost (mL)	1022.29±28.83	1081.57±197.61	939.28±234.42	0.014^*^
PHRR (%)	38.61±2.40	26.57±12.35	55.47±11.22	<0.001^*^
RI (%)	50.84±6.08	27.91±24.82	82.94±52.39	<0.001^*^
FLIS	3 (2,5.95)	3 (2,6)	3 (2,5.7)	0.497
Pathological characteristics				
Inflammation grade				0.767
A0	0 (0.00)	0 (0.00)	0 (0.00)	
A1	6 (10.00)	3 (8.57)	3 (12.00)	
A2	22 (36.67)	12 (34.29)	10 (40.00)	
A3	32 (53.33)	20 (57.14)	12 (48.00)	
Fibrosis stage				0.134
F0-1	16 (26.67)	9 (25.71)	7 (28.00)	
F2	9 (15.00)	3 (8.57)	6 (24.00)	
F3	15 (25.00)	12 (34.29)	3 (12.00)	
F4	20 (33.33)	11 (31.43)	9 (36.00)	
Steatosis grade				0.844
S0	25 (41.67)	14 (40.00)	11 (44.00)	
S1	33 (55.00)	20 (57.14)	13 (52.00)	
S2-3	2 (3.33)	1 (2.86)	1 (4.00)	
T1 mapping parameters				
T1-pre (ms)	992.65±10.05	1003.72±60.96	977.15±58.88	0.195
T1-HBP (ms)	485.67±8.52	496.01±588.27	471.20±74.35	0.153
Δ%	51.06±0.73	50.54±5.56	51.79±5.86	0.405

Data are represented in mean ± SD or medians with interquartile ranges, or frequency (%); And Data were evaluated by independent t test or Mann-Whitney U test for continuous variables and the Chi-square test or Fisher’s exact test or Wilcoxon rank sum test for categorical variables; ^*^ referred to P<0.05; RI, regeneration index; PHRR, parenchymal hepatic resection rate; BMI, body mass index; ALT, Alanine aminotransferase; AST, Aspartate aminotransferase; ALP, alkaline phosphatase; GGT, γ-Glutamyl Transferase; ALB, albumin; TBIL, total bilirubin; DBIL, direct bilirubin; HGB, hemoglobin; PLT, Platelet count; PT, Prothrombin time; INR, international normalized ratio; ALBI, albumin-bilirubin grade; LVpre: volume of preoperative future remnant liver; LVpost: volume of postoperative remnant liver, RI, regeneration index; PHRR, parenchymal hepatic resection rate; T1-pre, T1 relaxation time of the liver before gadoxetic acid injection; T1-HBP, T1 relaxation time of the liver 20 min after gadoxetic acid injection; Δ%, the reduction rate of T1 relaxation time.

When considering T1 mapping parameters and FLIS, detailed values of T1_pre_ (ms), T1_HBP_ (ms), Δ% and FLIS of all the 60 patients included in the study was listed in **Supplementary Table S1**. The ICC value of the two radiologists for T1_pre_, T1_HBP_, Δ% and FLIS were 0.881 (95% CI, 0.808-0.927), 0.920 (95% CI, 0.895-0.936), 0.903 (95% CI, 0.889-0.918) and 0.933 (95% CI, 0.921-0.946) respectively. No significant difference of T1 mapping parameters and FLIS (all *P*>0.05) manifested between patients receiving minor and major hepatectomy (**Table 2**). Due to the excellent agreement in ICCs of FLIS, for simplicity, only the FLIS assessed by the more experienced radiologist 2 were used in the analyses.

**Table 2:** Results of the univariate analysis of correlations between the regeneration index and preoperative variables.

Variables	Total patients (n=60)		Minor hepatectomy (n=35)		Major hepatectomy (n=25)	
	Correlation coefficient	*P* value	Correlation coefficient	*P* value	Correlation coefficient	*P* value
Age (years)	-0.176	0.178	-0.116	0.333	-0.128	0.542
Gender	0.022	0.870	0.148	0.395	0.061	0.774
PHRR (%)	0.770	<0.001^*^	0.444	0.007^*^	0.609	0.001^*^
BMI	0.089	0.504	0.196	0.267	-0.120	0.566
ALB (g/L)	0.316	0.014^*^	0.199	0.251	0.480	0.015^*^
ALT (IU/L)	0.097	0.464	-0.289	0.097	0.276	0.181
AST (IU/L)	0.183	0.163	-0.320	0.061	0.182	0.384
ALP (IU/L)	0.067	0.611	-0.041	0.816	-0.065	0.759
GGT (IU/L)	0.095	0.472	-0.290	0.091	-0.324	0.115
TBIL (umol/L)	-0.005	0.970	-0.248	0.151	-0.131	0.532
DBIL (umol/L)	-0.031	0.816	-0.189	0.278	-0.158	0.452
HGB (g/L)	-0.137	0.302	-0.148	0.404	-0.218	0.296
PLT (10^9/L)	0.242	0.065	0.307	0.077	0.104	0.622
PT (s)	0.125	0.346	-0.090	0.614	-0.290	0.159
INR	0.048	0.716	-0.067	0.705	-0.148	0.479
HbsAg	-0.026	0.846	0.060	0.732	-0.273	0.186
HBeAg	-0.203	0.120	-0.194	0.264	–	–
ALBI grade	-0.014	0.914	-0.162	0.353	–	–
Type of partial hepatectomy	0.671	<0.001^*^	–	–		
FLIS	0.300	0.020^*^	0.484	0.003^*^	0.275	0.076
LVpre (mL)	-0.817	<0.001^*^	-0.578	<0.001^*^	-0.603	0.001^*^
T1-pre (ms)	-0.467	<0.001^*^	-0.378	0.025^*^	-0.575	0.003^*^
T1-HBP(ms)	-0.592	<0.001^*^	-0.725	<0.001^*^	-0.430	0.032^*^
Δ%	0.395	0.002^*^	0.587	<0.001^*^	0.081	0.701

^*^ referred to *P*<0.05; RI, regeneration index; PHRR, parenchymal hepatic resection rate; BMI, body mass index; ALT, Alanine aminotransferase; AST, Aspartate aminotransferase; ALP, alkaline phosphatase; GGT, γ-Glutamyl Transferase; ALB, albumin; TBIL, total bilirubin; DBIL, direct bilirubin; HGB, hemoglobin; PLT, Platelet count; PT, Prothrombin time; INR, international normalized ratio; ALBI, albumin-bilirubin grade; LVpre: volume of preoperative future remnant liver; LVpost: volume of postoperative remnant liver; T1-pre, T1 relaxation time of the liver before gadoxetic acid injection; T1-HBP, T1 relaxation time of the liver 20 min after gadoxetic acid injection; Δ%, the reduction rate of T1 relaxation time.

### The relations between T1 mapping parameters, FLIS and liver fibrosis

**Figure 2:**
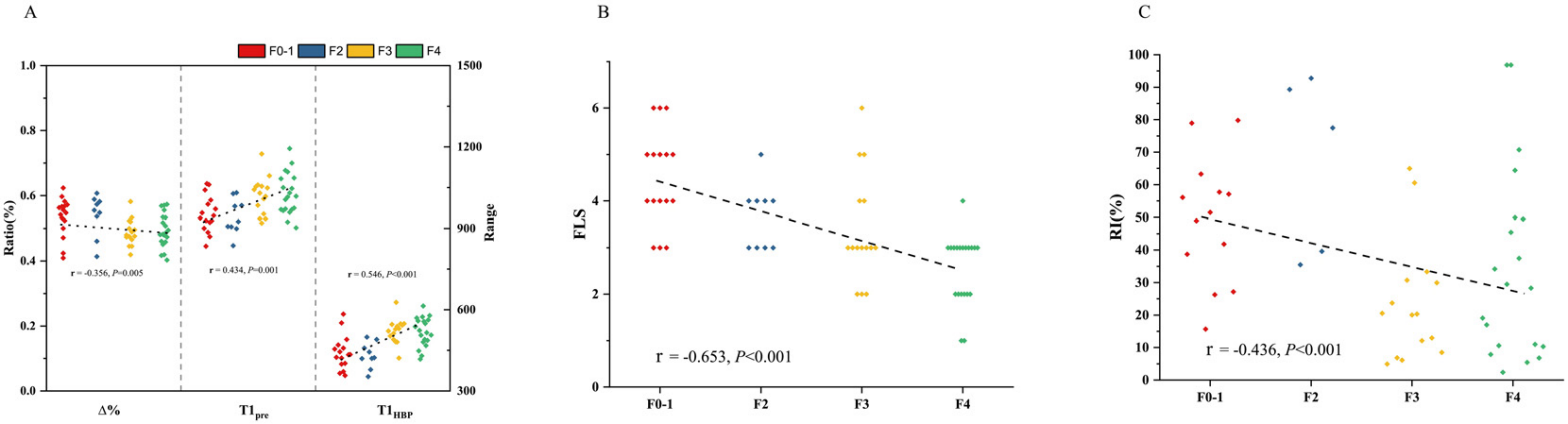
The relation between T1 mapping parameters and fibrosis stage **(A)**, FLIS and fibrosis stage **(B)**, fibrosis stage and RI **(C)** in total patients. RI, regeneration index; T1pre, T1 relaxation time of the liver before gadoxetic acid injection; T1HBP, T1 relaxation time of the liver 20 min after gadoxetic acid injection; Δ%, the reduction rate of T1 relaxation time; FLIS a functional liver imaging score.

**Figure 3:**
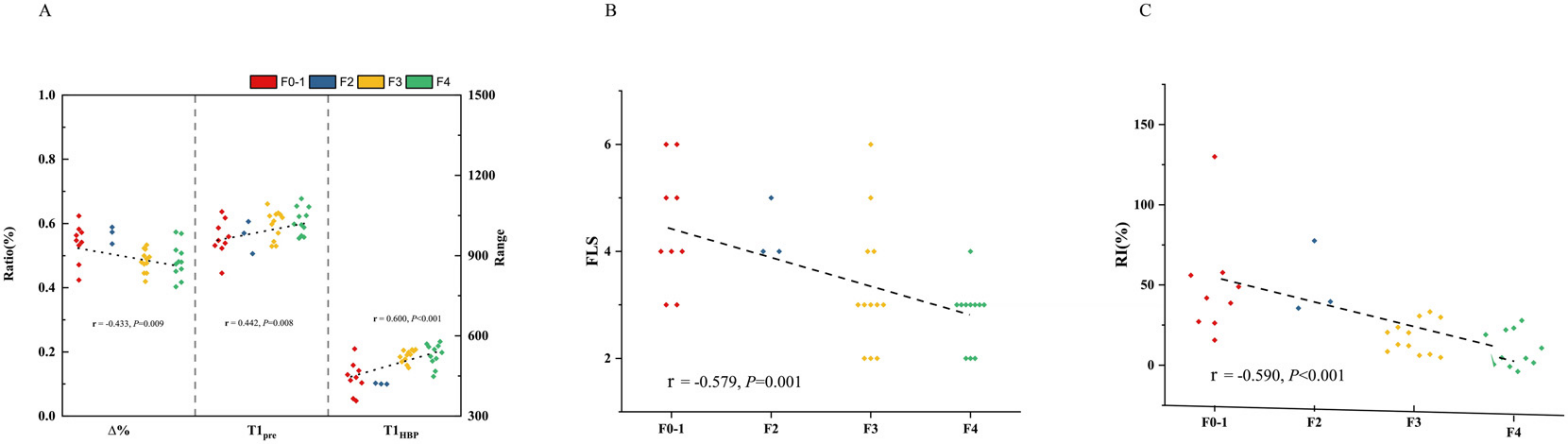
The relation between T1 mapping parameters and fibrosis stage **(A)**, FLIS and fibrosis stage **(B)**, fibrosis stage and RI **(C)** in patients undergoing minor hepatectomy. RI, regeneration index; T1pre, T1 relaxation time of the liver before gadoxetic acid injection; T1HBP, T1 relaxation time of the liver 20 min after gadoxetic acid injection; Δ%, the reduction rate of T1 relaxation time; FLIS, a functional liver imaging score.

**Figure 4:**
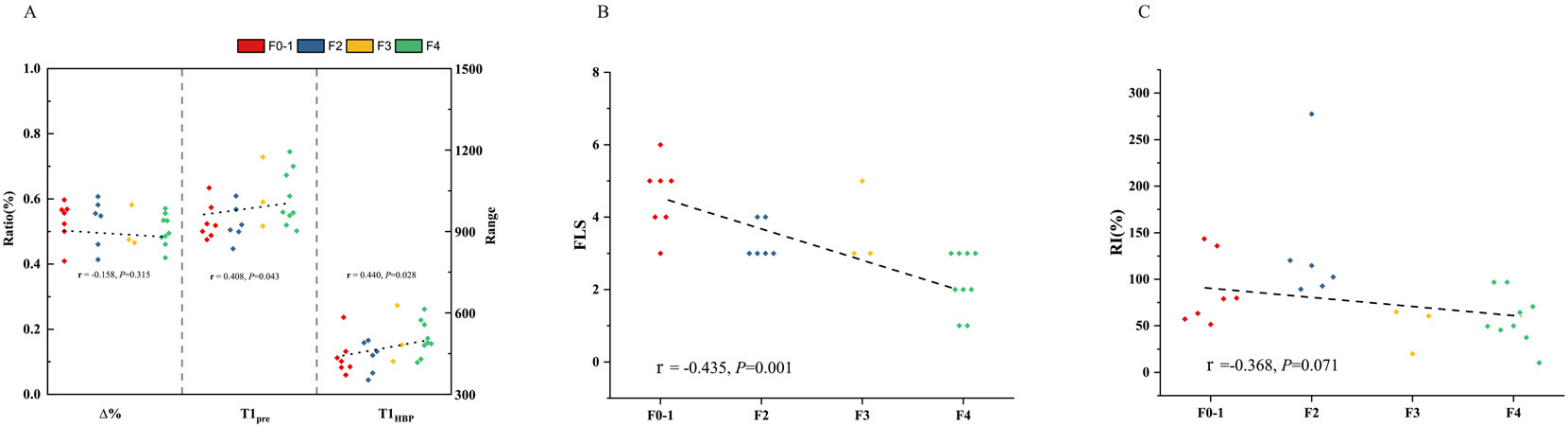
The relation between T1 mapping parameters and fibrosis stage **(A)**, FLIS and fibrosis stage **(B)**, fibrosis stage and RI **(C)** in patients undergoing major hepatectomy. RI, regeneration index; T1pre, T1 relaxation time of the liver before gadoxetic acid injection; T1HBP, T1 relaxation time of the liver 20 min after gadoxetic acid injection; Δ%, the reduction rate of T1 relaxation time; FLIS, a functional liver imaging score.

In all the patients, T1_pre_ and T1_HBP_ manifested a significant tendency of positive correlation with fibrosis stage (r = 0.434, *P* =0.001 and r = 0.546, *P* < 0.001, respectively, **Figure 2A**), and a significant tendency of negative correlation also showed between Δ% and fibrosis stage (r = -0.356, *P* =0.005, **Figure 2A**). FLIS also showed significant correlation with fibrosis stage (r = -0.653, *P <*0.001, **Figure 2B**). Meanwhile, T1_pre_, T1_HBP,_ Δ% and FLIS also showed significant correlations with fibrosis stage (T1_pre_: r =0.442, *P*=0.008; T1_HBP_: r =0.600, *P*<0.001; Δ%: r =-0.433, *P*=0.009; FLIS, r =-0.579, *P*=0.001, **Figures 3A, B**) in the patients undergoing minor hepatectomy. While in the patients undergoing major hepatectomy, moderate correlation showed between T1_pre_ and fibrosis stage (r = 0.408, *P*=0.043, **Figure 4A**), T1_HBP_ and fibrosis stage (r = 0.440, *P*=0.028, **Figure 4A**), FLIS and fibrosis stage (r =-0.435, *P*=0.001, **Figure 4B**).

### The relations between liver fibrosis and RI

In the total patients, fibrosis stage and RI manifested a statistically significant negative correlation (r = -0.436, *P<*0.001, **Figure 2C**). Besides, a significant correlation occurred between fibrosis stage and RI (r = -0.590, *P<*0.001, **Figure 3C**) in the patients undergoing minor hepatectomy. However, fibrosis stage did not show a significant negative correlation with RI (r =-0.368, *P*= 0.071, **Figure 4C**) in the patients undergoing major hepatectomy.

### The relations between T1 mapping parameters, FLIS and hepatic steatosis

Whether in the total patients or subgroups undergoing minor or major hepatectomy, T1_pre_ manifested significant correlation with steatosis grade (total patients: r = 0.415, *P* =0.001, **Supplementary Figure S1A**; patients undergoing minor hepatectomy: r = 0.470, *P* =0.004, **Supplementary Figure S2A**; patients undergoing major hepatectomy: r = 0.413, *P* =0.040, **Supplementary Figure S3A**), however, T1_HBP,_ Δ% and FLIS did not show significant correlation with steatosis grade (all *P*>0.05, **Supplementary Figures S1–S3A, S1–S3B**).

### The relations between hepatic steatosis and RI

Whether in the total patients or subgroups undergoing minor hepatectomy, significant correlation was observed between steatosis grade and RI (total patients: r = -0.338, *P* =0.008, **Supplementary Figure S1C**; patients undergoing minor hepatectomy: r = -0.403, *P* =0.004, **Supplementary Figure S2C**). However, no statistically significant correlation was observed between steatosis grade and RI in the patients undergoing major hepatectomy (r = -0.188, *P* =0.369, **Supplementary Figure S3C**).

### The relations between T1 mapping parameters and RI

#### Total patients

**Figure 5:**
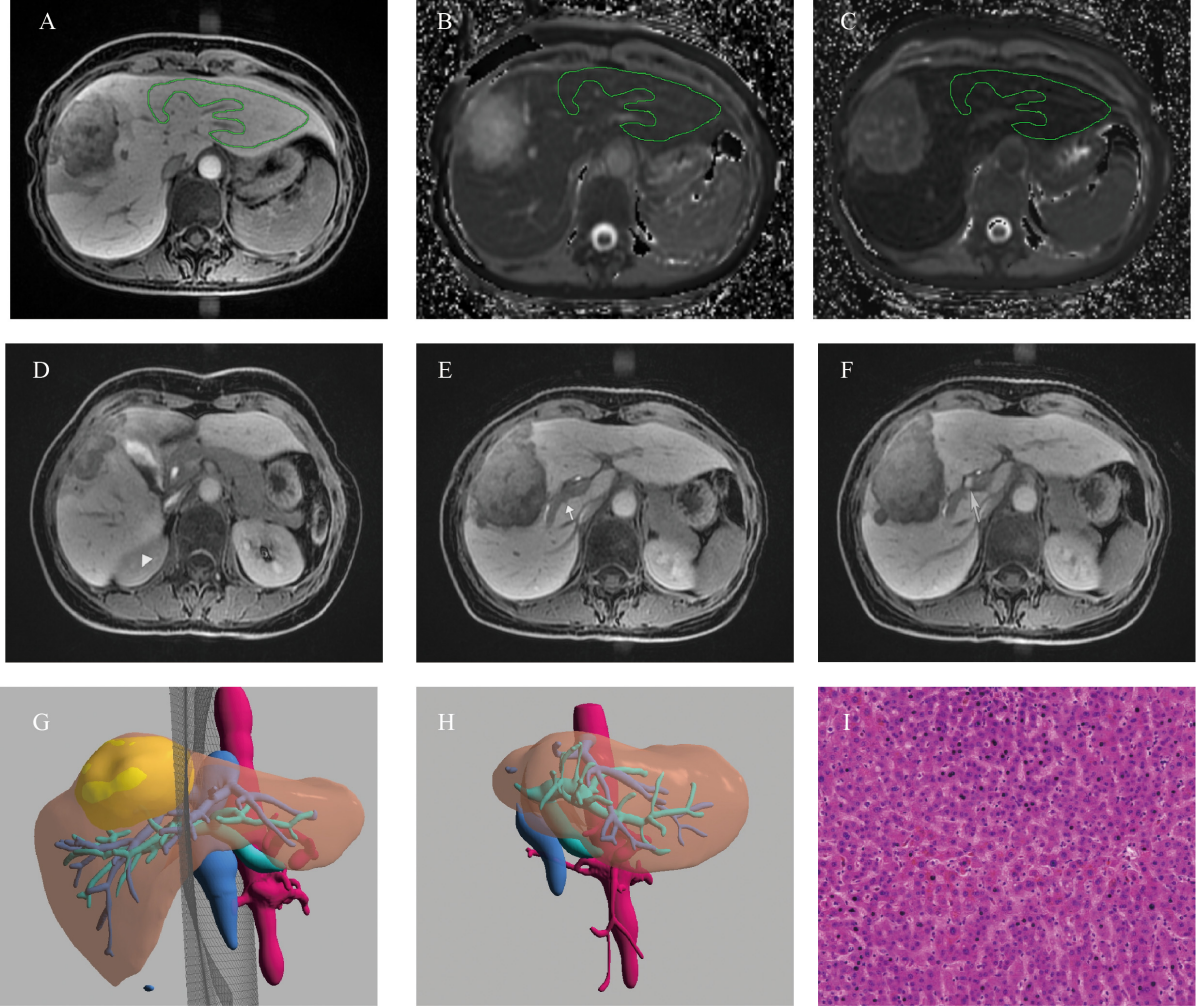
Example of a 52-years old female patients with HCC. **(A)** The ROI placement of live parenchyma based on HBP, **(B)** the T1pre was 921.79 ms, **(C)** the T1_HBP_ was 371.71 ms, and Δ% was 59.73%; **(D)**The signal intensity of liver parenchyma was isointense to the right kidney (triangle, score 1); **(E)** biliary contrast excreted into the common bile duct (dovetail arrow, score 2); **(F)** the portal vein (arrow) demonstrated hypointensity relative to thehepatic parenchyma (score 2), hence, the functional liver imaging score (FLIS) was 5; **(G)**The axial cross section image of the preoperative simulated surgical tangent; **(H)**The axial image of the actual remnant liver on the sixth month after surgery, and RI was 78.94%; **(I)** The fibrosis stage of lesion-free area was histopathologically proven to be F1 (original magnification, ×20); RI, regeneration index; T1pre, T1 relaxation time of the liver before gadoxetic acid injection; T1_HBP_, T1 relaxation time of the liver 20 min after gadoxetic acid injection; Δ%, The reduction rate of T1 relaxation time, FLIS, a functional liver imaging score.

In the total patients, in addition to PHRR (r = 0.770, *P*<0.001), ALB (r = 0.316, *P*=0.014), type of partial hepatectomy (r = 0.671, *P*<0.001) and LVpre(r = -0.817, *P*<0.001), T1pre(r = -0.467, *P<*0.001), T1_HBP_(r = -0.592, *P <*0.026) and Δ% (r = 0.395, *P* = 0.002), FLIS (r = 0.300, *P=*0.020) were also identified as significant predictors of RI in spearman relation analysis (**Table 2**, **Figure 5**).

Multicollinearity occurs If two variables with the strong intercorrelation were simultaneously included in multivariate analysis; hence, only one variable was included to avoid the occurrence of multicollinearity. Considering the strong intercorrelation between PHRR and LV_pre_ (r= -0.785, *P*<0.001, **Supplementary Figure S4A**), PHRR and type of partial hepatectomy (r= 0.794, *P*<0.001, **Supplementary Figure S4B**), LV_pre_ and type of partial hepatectomy (r= -0.785, *P*<0.001, **Supplementary Figure S4C**), T1_pre_ and T1_HBP_(r= 0.485, *P*<0.001, **Supplementary Figure S5A**), T1_HBP_ and Δ% (r= -0.834, *P*<0.001, **Supplementary Figure S5C**), FLIS and T1_pre_(r= -0.213, *P*=0.029, **Supplementary Figure S5D**), FLIS and T1_HBP_(r= -0.398, *P*<0.001, **Supplementary Figure S5E**), FLIS and Δ% (r=0.286, *P*=0.003, **Supplementary Figure S5F**). Accordingly, 9 independent models in multivariate analysis were tested (Model 1 included ALB, PHRR,T1pre and Δ%; Model 2 included ALB, PHRR and T1_HBP_; Model 3 included ALB, Type of partial hepatectomy, T1pre and Δ%; Model 4 included ALB, Type of partial hepatectomy and T1_HBP_; Model 5 included ALB, LVpre, T1pre and Δ%; Model 6 included ALB, LVpre and T1_HBP;_ Model 7 included ALB, LVpre and FLIS; Model 8 included ALB, Type of partial hepatectomy and FLIS; Model 9 included ALB, PHRR and FLIS), Model 1 achieved the highest adjusted R^2^ of 0.594. T1pre, T1_HBP_ and FLIS showed significantly negative or positive linear associations with RI in all the models (T1_pre_:Model 1, Standardized β = -0.346, *P*< 0.001; Model 3, Standardized β = -0.386, *P*<0.001; Model 5: Standardized β = -0.305, *P*=0.003; T1_HBP_: Model 2, Standardized β = -0.272, *P*=0.006; Model 4, Standardized β = -0.362, *P*<0.001; Model 6: Standardized β = -0.183, *P*= 0.005; FLIS: Model 7, Standardized β = 0.233, *P*=0.037; Model 8, Standardized β = 0.256, *P*=0.040; Model 9, Standardized β = 0.231, *P*=0.033, **Table 3**). The whole VIFs were under 10, and it meant that no multicollinearity occurred between variables in all the multivariate model.

**Table 3:** Results of the multivariate linear regressions with RI as the dependent variable in total patients, in patients undergoing minor hepatectomy, and in patients undergoing major hepatectomy.

	Covariates	Standardized β	*P* value	Adjusted R^2^ of the Model	VIF
**Total Patients** **(n=60)**	Model 1			0.594	
	ALB	0.096	0.278		1.034
	PHRR	0.570	<0.001^*^		1.151
	T1_pre_	-0.346	<0.001^*^		1.054
	Δ%	0.104	0.246		1.071
	Model 2			0.517	
	ALB	0.103	0.269		1.032
	PHRR	0.580	<0.001^*^		1.147
	T1_HBP_	-0.272	0.006^*^		1.113
	Model 3			0.495	
	Type of partial hepatectomy	0.479	<0.001^*^		1.057
	ALB	0.140	0.139		1.014
	T1_pre_	-0.386	<0.001^*^		1.033
	Δ%	0.188	0.048^*^		1.016
	Model 4			0.456	
	Type of partial hepatectomy	0.497	<0.001^*^		1.049
	ALB	0.146	0.138		1.013
	T1_HBP_	-0.362	<0.001^*^		1.036
	Model 5			0.501	
	LV_pre_	-0.565	<0.001^*^		1.435
	ALB	0.042	0.662		1.102
	T1_pre_	-0.305	0.003^*^		1.120
	Δ%	0.020	0.842		1.227
	Model 6			0.448	
	LV_pre_	-0569	<0.001^*^		1.422
	ALB	0.051	0.617		1.097
	T1_HBP_	-0.183	0.005^*^		1.321
	Model 7			0.462	
	LV_pre_	-0.561	<0.001		1.331
	ALB	0.001	0.992		1.086
	FLIS	0.233	0.037		1.312
	Model 8			0.372	
	Type of partial hepatectomy	0.443	<0.001		1.326
	ALB	0.093	0.383		1.062
	FLIS	0.256	0.040		1.390
	Model 9			0.490	
	PHRR	0.572	<0.001		1.250
	ALB	0.051	0.596		1.067
	FLIS	0.231	0.033		1.293
**Patients undergoing minor hepatectomy** **(n=35)**	Model 1			0.594	
	PHRR	0.324	0.010^*^		1.157
	T1_pre_	-0.180	0.140		1.189
	T1_HBP_	-0.549	<0.001^*^		1.340
	Model 2			0.609	
	PHRR	0.316	0.010^*^		1.161
	T1_pre_	-0.455	<0.001^*^		1.020
	Δ%	0.532	<0.001^*^		1.182
	Model 3			0.573	
	LV_pre_	-0.310	0.024^*^		1.350
	T1_pre_	-0.163	0.191		1.176
	T1_HBP_	-0.510	<0.001^*^		1.552
	Model 4			0.586	
	LV_pre_	-0.297	0.028		1.363
	T1_pre_	-0.420	0.001^*^		1.052
	Δ%	0.497	0.001^*^		1.378
	Model 5			0.492	
	LV_pre_	-0.474	0.001		1.069
	T1_pre_	-0.217	0.107		1.144
	FLIS	0.029	0.017		1.214
	Model 6			0.457	
	PHRR	0.434	0.002		1.060
	T1_pre_	-0.261	0.064		1.156
	FLIS	0.349	0.017		1.212
**Patients undergoing major hepatectomy** **(n=25)**	Model 1			0.223	
	PHRR	0.436	0.027^*^		1.047
	ALB (g/L)	-0.208	0.267		1.028
	T1_HBP_	-0.243	0.206		1.076
	Model 2			0.159	
	LV_pre_	-0.407	0.042^*^		1.311
	ALB (g/L)	0.189	0.333		1.035
	T1_HBP_	-0.136	0.537		1.347
	Model 3			0.194	
	LV_pre_	-0.342	0.140		1.469
	ALB	0.043	0.835		1.239
	T1_pre_	-0.338	0.114		1.237
	Δ%	0.127	0.550		1.303
	FLIS	0.080	0.700		1.240
	Model 4			0.261	
	PHRR	0.380	0.048		1.087
	ALB	0.050	0.800		1.240
	T1_pre_	-0.348	0.080		1.150
	Δ%	0.050	0.797		1.202
	FLIS	0.170	0.376		

^*^ referred to *P*<0.05; PHRR, parenchymal hepatic resection rate; LVpre: volume of preoperative future remnant liver; T1-pre, T1 relaxation time of the liver before gadoxetic acid injection; T1-HBP, T1 relaxation time of the liver 20 min after gadoxetic acid injection; Δ%, the reduction rate of T1 relaxation time.

#### Patients undergoing minor or major hepatectomy

Similarly, considering the strong intercorrelation between PHRR and LV_pre_ (r= -0.495, *P*=0.003, **Supplementary Figure S6A**), T1_HBP_ and Δ% (r= 0.879, *P*<0.001, **Supplementary Figure S6D**), T1_HBP_ and FLIS (r= -0.478, *P*=0.001, **Supplementary Figure S6F**), Δ% and FLIS (r=0.386, *P*=0.003, **Supplementary Figure S6G**) in patients undergoing minor hepatectomy, accordingly, 6 independent models in multivariate analysis were tested (Model 1 included PHRR, T1_pre_ and T1_HBP_; Model 2 included PHRR, T1_pre_ and Δ%; Model 3 included LV_pre_, T1_pre_ and T1_HBP_, T1_pre_ and Δ%; Model 4 included LV_pre_, T1_pre_ and Δ%; Model 5 included LV_pre_, T1_pre_ and FLIS; Model included PHRR, T1_pre_ and FLIS), T1_HBP_, Δ% and FLIS still showed significantly negative or positive linear associations with RI in all the models (T1_HBP_: Model 1, Standardized β = -0.549, *P*< 0.001; Model 3, Standardized β = -0.510, *P*<0.001; Δ%:Model 2: Standardized β = 0.532,*P<*0.003; Model 4, Standardized β = 0.497, *P*=0.001; FLIS: Model 5, Standardized β = 0.029, *P*=0.017; Model 6, Standardized β = 0.349, *P*=0.017, **Table 3**). And all the VIFs were under 10.

Meanwhile, in consideration of the strong intercorrelation between PHRR and LV_pre_ (r= -0.579, *P*=0.002, **Supplementary Figure S7A**), T1_pre_ and T1_HBP_ (r= 0.532, *P*=0.006, **Supplementary Figure S7B**) and T1_HBP_ and Δ% (r= -0.821, *P*<0.001, **Supplementary Figure S7D**), T1_HBP_ and FLIS(r= -0.340, *P*=0.028, **Supplementary Figure S7F**) in patients undergoing major hepatectomy, accordingly, 4 independent models in multivariate analysis were tested(Model 1 included ALB, PHRR and T1_HBP_; Model 2 included ALB, LV_pre_ and T1_HBP_; Model 3 included ALB, LV_pre_, T1_pre_, Δ% and FLIS; Model 4 included ALB, PHRR, T1_pre_, Δ% and FLIS), however, no T1 mapping parameters and FLIS (all *P >*0.05)showed a significantly linear association with RI.

## Discussion

This present study explored the utility of T1-mapping parameters of pre-contrast, 20-min hepatobiliary phase (HBP), FLIS for liver regeneration assessment in HCC patients undergoing hepatectomy. The results demonstrated that T1_pre_, T1_HBP_, Δ% and FLIS score were the significant preoperative biomarkers of liver regeneration whether in univariate or multivariable analysis. They may help optimize patient selection for hepatectomy, promote individualized therapy, and reduce the occurrence of potential complications. Meanwhile, T1 mapping parameters and FLIS were closely associated with the severity of liver fibrosis, which are of vital importance for LR assessment. Besides, T1_pre_ was also related to the severity of steatosis. However, for patients undergoing major hepatectomy, although T1 mapping parameters and FLIS were still related to liver fibrosis, they were not effective indicators of liver regeneration.

In line with our study, the increase of T1 relaxation times (T1_pre_) before contrast injection correspond with the stage of hepatic fibrosis, due to the fact that the progression of hepatic fibrosis is believed to be accompanied with to an increase in extracellular water and protein concentration, finally leading to the increase the T1 relaxation time of liver parenchyma (11, 26, 27). Moreover, along with the increasing degree of fibrosis, disrupted architecture, aberrant hepatocyte regeneration, increased extracellular constituents, and vascular changes of liver parenchyma would cause a decreased number of normally functioning hepatocytes, a decreasing trend in Oatps level and an increasing trend in Mrps level with disturbed transporting system (28–30). The uptake of Gd-EOB-DTPA in hepatocytes occurs via Oatps expressed at the sinusoidal membrane, and its biliary excretion occurs via Mrps at the canalicular membrane (31). Accordingly, with the progression of liver fibrosis, the uptake of Gd-EOB-DTPA in hepatocytes would be in decrease and the secretion would be in increase due to the decreasing Oatps expression and the increasing Mrps expression, finally resulting in the significant increase of T1 relaxation times (T1_HBP_) and the significant decrease of signal enhancement of parenchyma after Gd-EOB-DTPA injection. Hence, FLIS score, based on the signal enhancement of liver parenchyma after Gd-EOB-DTPA injection, was reasonable to be in decrease due to the decreasing Oatps expression, further negatively associated with the stage of liver fibrosis. Δ%, which was calculated as [(T1_pre_) – (T1_HBP_)]/(T1_HBP_) × 100%, showed significantly negative relation with the stage of liver fibrosis in this study. This may be explained that T1_pre_ increased by a lower degree than T1_HBP_ in the progression of liver fibrosis, which have been found in Sheng’s study [32]. In line with our study, previous studies (32, 33) has also found that T1_HBP_ was in an increasing trend while Δ% gradually decreased with the progression of liver fibrosis. Meanwhile, consistent with our study, steatosis grade had strong correlation with T1_pre_ (17). The potential reason was that hepatic steatosis would induce a mixture of water and fat, the fat would be generally out-of-phase with water, and the MR signal would be subtracted by the fat component, resulting in longer T1 relaxation time (T1_pre_) (13, 14). However, compared with that Oatps expression was in decrease and Mrps expression was in increase due to liver fibrosis, hepatic steatosis was often along with the descending expression of Oatps and unchanged or even decreasing expression of Mrps (34, 35). Accordingly, the uptake of Gd-EOB-DTPA in hepatocytes would be in decrease and the secretion decelerated. Thus, the clearance rate of Gd-EOB-DTPA in hepatocytes due to hepatic steatosis was slower than that due to liver fibrosis, finally leading to that the increase of T1 relaxation times (T1_HBP_) and the decrease of signal enhancement of parenchyma after Gd-EOB-DTPA injection due to hepatic steatosis was by a lower degree than that due to liver fibrosis. This was the potential reason why T1_HBP,_ Δ% and FLIS showed significant correlation with fibrosis stage but no statistical correlation with steatosis grade. Similar to our study, Yang et al. found only T1_pre_ was significantly associated with liver steatosis at multiple regression analysis while T1_HBP_ and Δ% was not. Moreover, consistent with our study, the severity of liver fibrosis was associated with the capacity of liver regeneration (36, 37). Fibrosis overrides liver regeneration through differential recruitment of pro-regenerative CXCR7-Id1 versus pro-fibrotic FGFR1-CXCR4 angiocrine pathways in vascular niche (38). When overstimulated, selective CXCR4 activation in liver sinusoidal endothelial cells could abrogate regeneration. Meanwhile, evidence (39) has also proven that liver fibrosis would cause the shortening of hepatocyte telomeres, leading to a decrease in the number of cell divisions of primary human cells, finally restraining liver regeneration. Besides, hepatic steatosis would cause lipid overloading, which is associated with endoplasmic reticulum stress and oxidative stress, leading to delayed hepatocyte DNA replication, finally resulting in the impair of liver regeneration (40). Hence, it is reasonable that T1 mapping parameters and FLIS are closely associated with liver regeneration. Although T1 mapping and FLIS on Gd-EOB-DTPA-enhanced MRI require the injection of contrast agent, reduced or absent hepatocyte function is the major pathophysiologic impairment in severely fibrotic and cirrhotic patients with low capacity of liver regeneration. T1 mapping on Gd-EOB-DTPA-enhanced MRI could directly reflect hepatocyte function through effectively assessing the ability of hepatocytes to uptake Gd-EOB-DTPA, while IVIM-DWI and MRE can only indirectly assess hepatocyte function by reflecting liver stiffness, the diffusion of water molecules and the liver blood perfusion (41). Hence, it is of pivotal importance for the use of T1 mapping parameters and FLIS based on Gd-EOB-DTPA-enhanced MRI in liver regeneration assessment for that it could directly reflect hepatocyte function. However, there is lack of direct comparisons whether T1 mapping on Gd-EOB-DTPA-enhanced MRI is superior over IVIM-DWI and MRE in the liver regeneration estimation, and it is warranted to go on comparison in the further study.

However, in the patients undergoing major hepatectomy, not only hepatic steatosis, liver fibrosis was also not associated with liver regeneration, which was in line with finding from Jang’s study (7). We hypothesize that the factors affecting hepatic function are of secondary importance for liver regeneration after a large liver resection (where the liver remnant is relatively small). Compared with minor hepatectomy, major hepatectomy often means the increase in the ratio of blood flow to the remnant liver and relatively more remnant liver cells, which is in need to preserve appropriate liver function and support the metabolic needs. These changes may lead to a greater concentration of cytokines and the more effective hepatic proliferation, finally promoting liver regeneration (2, 42). Accordingly, it is conceivable that although T1_pre_, T1_HBP_ and FLIS showed moderate correlation with liver fibrosis stage, significant correlation was observed between T1_pre_ and hepatic steatosis, no T1 mapping parameters and FLIS were related to liver regeneration in patients undergoing major hepatectomy.

All of T1 mapping parameters in present study were not associated with grading inflammation (The details are shown in **Supplementary Table S2**), which was not in line with previous study (27). It may be explained by that all the patients in present study were confirmed as HCC, their inflammation grade was more serious and the majority (90%) was in inflammation grade 2-3.

Larger PHRR are often accompanied with larger resection volume and smaller LV_pre_ (20). Thus, there is reasonable intercorrelation between PHRR, LV_pre_ and type of hepatectomy. PHRR, LV_pre_ and the type of hepatectomy were associated with LR in previous studies (43, 44). Compared with PHRR, the type of hepatectomy and LV_pre_ overlooked the considerable interpatient variability in the size of the various liver segments. And it may be the possible cause that the model including PHRR achieved the better adjusted R^2^ whether in the total patient or patient undergoing major or minor hepatectomy. In the present study, ALB was a relevant factor affecting liver regeneration, which was consistent with previous studies (21, 45). It is likely that liver fibrosis, accompanied by the damage of liver regeneration, could cause a reduction in normal hepatocytes, finally resulting in lower ALB levels (46).

This present study is based on saturation method using adaptive recovery times for T1 mapping sequence (SMART1Map), rather than modifed lock-locker inversion recovery (MOLLI). Compared with MOLLI, SMART1Map is less sensitive to imaging parameters, such as T2 times and magnetization transfer, does not require correction (47, 48). Therefore, T1 mapping parameters based on SAMRT1Map for liver regeneration assessment are more stable and reliable. Gadoxetic acid disodium is a liver specific contrast agent which has reported to achieve comparable diagnostic performance with extra cellular contrast agent but provided additional hepatobiliary phase (HBP). For liver regeneration assessment, T1_HBP_ could reflect both water molecules in liver parenchyma, which could also be evaluated by T1pre, and Oatp1a1 level with disturbed transporting system, and Δ% were calculated as (T1_pre_-T1_HBP_)/T1_pre_ × 100%. FLIS takes into account three features of gadoxetic acid–enhanced MRI of the liver: enhancement quality, rate of biliary contrast excretion, and persistence of signal intensity in the portal vein. Thus, it is reasonable for the existence of intercorrelation between T1_pre_ and T1_HBP_, T1_HBP_ and Δ%, T1_pre_ and FLIS, T1_HBP_ and FLIS, Δ% and FLIS.

When considering the potential clinical complication of T1 mapping parameters and FLIS score, they may serve as useful tips tools to inform a potential paradigm shift in treating HCC patients. Specifically, for HCC patients exhibiting higher T1_pre_ and T1_HBP_ values, coupled with lower Δ% and FLIS values, there is a greater tendency for them to have a higher degree of liver fibrosis and hepatic steatosis, a reduced capacity for liver regeneration, thereby increasing the likelihood of developing postoperative liver failure. These patients may be considered as more suitable candidates for alternative treatment approaches rather than upfront surgery. For instance, along with the higher T1_pre_ and T1_HBP_ values, coupled with lower Δ% and FLIS values, the progression of liver fibrosis and the decline of liver regeneration capacity could alter hepatic immune microenvironment, leading to the presence of tumor-infiltrating lymphocytes such as CD8^+^and CD4^+^T cells, and the upregulation of exhaustion markers such as PD-L1, and CTLA-4 (49, 50). Immune checkpoint inhibition, such as anti-CTLA-4 therapy and anti-PD-L1 therapy, may be new treatment option. Due to the relatively small sample size and the nature of exploratory research without uniform threshold to distinguish low regeneration capacity from high regeneration capacity, no threshold of T1 mapping parameters and FLIS score were explored to distinguish regeneration capacity and guide clinical interventions, and it is warranted in the further study.

## Limitation

Our study has several limitations. Firstly, the relatively small size of the retrospective study population based on a single research institution limit the quality of evidence that no T1 mapping parameters and FLIS score were associated with liver regeneration for patients undergoing major hepatectomy. In the subsequent research, prospective studies with a larger sample based on multi-institution are warranted to further verify this tendency and provide higher quality evidence. In order to address the variability and ensure the reliability of the research, the standardize scanning protocols and standardized training for participating researchers would be used to reduce systemic errors and variability between different institution, and the influence of different institution would be taken into account as a covariate in statistical analysis. Secondly, due to the retrospective study, the non-uniform interval between postoperative CT image and surgery in the range of 1.5–11.5 months. In fact, due to that it is still unclear when regeneration is complete, the postoperative course of liver regeneration assessment was an inconsistent time such as 1–6 months (51). Besides, it has proven that the first week after surgery is the period with high speed in liver regeneration, and then the speed of regeneration slows down (52, 53). In our study, for patients undergoing multiple postoperative CT scan, the difference of RIs based on the multiple CT images ranged of 0.49%-19.55% (mean: 8.78%). Thus, the non-uniform interval between postoperative CT image and surgery in this present study was within limits of acceptability. A prospective study with uniform time interval for postoperative follow-up CT is warranted in the future. Thirdly, it is better to measure the preoperative volume using the immediate postsurgical CT. However, due to the respective nature, the patient usually underwent an abdominal plain CT scan after immediate hepatectomy. It is difficult to remove the hepatic portal vein, hepatic vein, and their main branches. Similar to previous studies (7, 20, 54), the present study used the previous CT image to calculate the preoperative volume, In the subsequent research, it is better to go on an immediate postsurgical measurement based on prospective nature. Besides, it is better to assess the fibrosis stage based on the preoperative remnant liver. However, it is unreasonable to go on liver biopsy of preoperative remnant liver, according to the previous study (7), surgical specimen was used for fibrosis stage assessment. In the subsequent prospective research, additional evaluation using US shear-wave elastography or acoustic radiation force impulse imaging can be done for liver fibrosis assessment. Finally, due to the retrospective nature, no comparison went on between T1 mapping with the alternative noninvasive methods included indocyanine green (ICG) clearance (55), MRE (7) and IVIM (56) for liver regeneration evaluation. In the subsequent research, alternative noninvasive methods for comparison are in need.

## Conclusion

In conclusion, T1pre, T1_HBP_, Δ% values and FLIS score of Gd-EOB-DTPA-enhanced MRI could be noninvasive imaging indicators for liver regeneration assessment, expect for patients undergoing major hepatectomy.

## Data Availability

The raw data supporting the conclusions of this article will be made available by the authors, without undue reservation.

## References

[1] Sung H Ferlay J Siegel RL Laversanne M Soerjomataram I Jemal A. Global cancer statistics 2020: GLOBOCAN estimates of incidence and mortality worldwide for 36 cancers in 185 countries. CA Cancer J Clin. (2021) 71:209–49. doi: 10.3322/caac.21660 33538338

[2] Inoue Y Fujii K Ishii M Kagota S Tomioka A Hamamoto H. Volumetric and functional regeneration of remnant liver after hepatectomy. J Gastrointest Surg. (2019) 23:914–21. doi: 10.1007/s11605-018-3985-5 30264387

[3] Cieslak KP Runge JH Heger M Stoker J Bennink RJ van Gulik TM. New perspectives in the assessment of future remnant liver. Dig Surg. (2014) 31:255–68. doi: 10.1159/000364836 25322678

[4] Marrone G Shah VH Gracia-Sancho J. Sinusoidal communication in liver fibrosis and regeneration. J Hepatol. (2016) 65:608–17. doi: 10.1016/j.jhep.2016.04.018 PMC4992446 27151183

[5] Allaire M Gilgenkrantz H. The impact of steatosis on liver regeneration. Horm Mol Biol Clin Investig. (2018) 41. doi: 10.1515/hmbci-2018-0050 30462610

[6] Li YT Cercueil JP Yuan J Chen W Loffroy R Wáng YX. Liver intravoxel incoherent motion (IVIM) magnetic resonance imaging: a comprehensive review of published data on normal values and applications for fibrosis and tumor evaluation. Quant Imaging Med Surg. (2017) 7:59–78. doi: 10.21037/qims.2017.02.03 28275560 PMC5337188

[7] Jang S Lee JM Lee DH Joo I Yoon JH Chang W. Value of MR elastography for the preoperative estimation of liver regeneration capacity in patients with hepatocellular carcinoma. J Magn Reson Imaging. (2017) 45:1627–36. doi: 10.1002/jmri.25517 27859840

[8] Gao Y Zheng J Liang P Tong M Wang J Wu C. Liver fibrosis with two-dimensional US shear-wave elastography in participants with chronic hepatitis B: A prospective multicenter study. Radiology. (2018) 289:407–15. doi: 10.1148/radiol.2018172479 30040048

[9] Ramos Rubio E Llado Garriga L. Usefulness of pre-surgical biopsy in selecting patients with hepatocellular carcinoma for liver transplant. Cir Esp. (2010) 87:133–8. doi: 10.1016/j.ciresp.2009.11.026 20074710

[10] Shin HJ Yoon H Kim MJ Han SJ Koh H Kim S. Liver intravoxel incoherent motion diffusion-weighted imaging for the assessment of hepatic steatosis and fibrosis in children. World J Gastroenterol. (2018) 24:3013–20. doi: 10.3748/wjg.v24.i27.3013 PMC6054952 30038468

[11] Li Z Sun J Hu X Huang N Han G Chen L. Assessment of liver fibrosis by variable flip angle T1 mapping at 3.0T. J Magn Reson Imaging. (2016) 43:698–703. doi: 10.1002/jmri.25030 26267123

[12] Xu XY Wang WS Zhang QM Li JL Sun JB Qin TT. Performance of common imaging techniques vs serum biomarkers in assessing fibrosis in patients with chronic hepatitis B: A systematic review and meta-analysis. World J Clin cases. (2019) 7:2022–37. doi: 10.12998/wjcc.v7.i15.2022 PMC6695542 31423434

[13] Ahn JH Yu JS Park KS Kang SH Huh JH Chang JS. Effect of hepatic steatosis on native T1 mapping of 3T magnetic resonance imaging in the assessment of T1 values for patients with non-alcoholic fatty liver disease. Magn Reson Imaging. (2021) 80:1–8. doi: 10.1016/j.mri.2021.03.015 33798658

[14] Mozes FE Tunnicliffe EM Pavlides M Robson MD. Influence of fat on liver T1 measurements using modified Look-Locker inversion recovery (MOLLI) methods at 3T. J Magn Reson Imaging. (2016) 44:105–11. doi: 10.1002/jmri.25146 PMC4982078 26762615

[15] Kim JE Kim HO Bae K Choi DS Nickel D. T1 mapping for liver function evaluation in gadoxetic acid-enhanced MR imaging: comparison of look-locker inversion recovery and B(1) inhomogeneity-corrected variable flip angle method. Eur Radiol. (2019) 29:3584–94. doi: 10.1007/s00330-018-5947-4 30903328

[16] Li J Liu H Zhang C Yang S Wang Y Chen W. Native T1 mapping compared to ultrasound elastography for staging and monitoring liver fibrosis: an animal study of repeatability, reproducibility, and accuracy. Eur Radiol. (2020) 30:337–45. doi: 10.1007/s00330-019-06335-0 31338650

[17] Yang R Chen Z Pan J Yang S Hu F. Non-contrast T1ρ dispersion versus Gd-EOB-DTPA-enhanced T1mapping for the risk stratification of non-alcoholic fatty liver disease in rabbit models. Magn Reson Imaging. (2024) 107:130–7. doi: 10.1016/j.mri.2024.01.013 38278311

[18] Lee HJ Hong SB Lee NK Kim S Seo HI Kim DU. Validation of functional liver imaging scores (FLIS) derived from gadoxetic acid-enhanced MRI in patients with chronic liver disease and liver cirrhosis: the relationship between Child-Pugh score and FLIS. Eur Radiol. (2021) 31:8606–14. doi: 10.1007/s00330-021-07955-1 33881570

[19] Aslan S Eryuruk U Tasdemir MN Cakir IM. Determining the efficacy of functional liver imaging score (FLIS) obtained from gadoxetic acid-enhanced MRI in patients with chronic liver disease and liver cirrhosis: the relationship between Albumin-Bilirubin (ALBI) grade and FLIS. Abdom Radiol (NY). (2022) 47:2325–34. doi: 10.1007/s00261-022-03557-7 35672474

[20] Kele PG de Boer M van der Jagt EJ Lisman T Porte RJ. Early hepatic regeneration index and completeness of regeneration at 6 months after partial hepatectomy. Br J Surg. (2012) 99:1113–9. doi: 10.1002/bjs.8807 22696005

[21] Zhang T Li Q Wei Y Yao S Yuan Y Deng L. Preoperative evaluation of liver regeneration following hepatectomy in hepatocellular carcinoma using magnetic resonance elastography. Quant Imaging Med Surg. (2022) 12:5433–51. doi: 10.21037/qims-22-306 PMC9703107 36465825

[22] Bedossa P Poynard T. An algorithm for the grading of activity in chronic hepatitis C. The METAVIR Cooperative Study Group. Hepatology. (1996) 24:289–93. doi: 10.1002/hep.510240201 8690394

[23] Scheuer PJ. Classification of chronic viral hepatitis: a need for reassessment. J Hepatol. (1991) 13:372–4. doi: 10.1016/0168-8278(91)90084-O 1808228

[24] Huang Z Zhou J Lu X Zhang T Xu S Jin J. How does liver steatosis affect diagnostic performance of 2D-SWE.SSI: assessment from aspects of steatosis degree and pathological types. Eur Radiol. (2021) 31:3207–15. doi: 10.1007/s00330-020-07288-5 33119813

[25] Bastati N Beer L Mandorfer M Poetter-Lang S Tamandl D Bican Y. Does the functional liver imaging score derived from gadoxetic acid-enhanced MRI predict outcomes in chronic liver disease? Radiology. (2020) 294:98–107. doi: 10.1148/radiol.2019190734 31743083

[26] Hoad CL Palaniyappan N Kaye P Chernova Y James MW Costigan C. A study of T_1_ relaxation time as a measure of liver fibrosis and the influence of confounding histological factors. NMR BioMed. (2015) 28:706–14. doi: 10.1002/nbm.3299 25908098

[27] von Ulmenstein S Bogdanovic S Honcharova-Biletska H Blümel S Deibel AR Segna D. Assessment of hepatic fibrosis and inflammation with look-locker T1 mapping and magnetic resonance elastography with histopathology as reference standard. Abdom Radiol (NY). (2022) 47:3746–57. doi: 10.1007/s00261-022-03647-6 PMC9560941 36038643

[28] Besa C Bane O Jajamovich G Marchione J Taouli B. 3D T1 relaxometry pre and post gadoxetic acid injection for the assessment of liver cirrhosis and liver function. Magn Reson Imaging. (2015) 33:1075–82. doi: 10.1016/j.mri.2015.06.013 26119422

[29] Ogasawara K Terada T Katsura T Hatano E Ikai I Yamaoka Y. Hepatitis C virus-related cirrhosis is a major determinant of the expression levels of hepatic drug transporters. Drug Metab Pharmacokinet. (2010) 25:190–9. doi: 10.2133/dmpk.25.190 20460825

[30] Lagadec M Doblas S Giraudeau C Ronot M Lambert SA Fasseu M. Advanced fibrosis: Correlation between pharmacokinetic parameters at dynamic gadoxetate-enhanced MR imaging and hepatocyte organic anion transporter expression in rat liver. Radiology. (2015) 274:379–86. doi: 10.1148/radiol.14140313 25289480

[31] Kimura Y Sato S Hitomi E Ohyama M Adachi K Inagaki Y. Coexpression of organic anion-transporting polypeptides 1B3 and multidrug-resistant proteins 2 increases the enhancement effect of gadolinium-ethoxybenzyl-diethylenetriamine pentaacetic acid on hepatocellular carcinoma in magnetic resonance imaging. Hepatol Res. (2014) 44:327–37. doi: 10.1111/hepr.2014.44.issue-3 23607695

[32] Sheng RF Wang HQ Yang L Jin KP Xie YH Fu CX. Assessment of liver fibrosis using T1 mapping on Gd-EOB-DTPA-enhanced magnetic resonance. Dig Liver Dis. (2017) 49:789–95. doi: 10.1016/j.dld.2017.02.006 28237298

[33] Pan S Wang XQ Guo QY. Quantitative assessment of hepatic fibrosis in chronic hepatitis B and C: T1 mapping on Gd-EOB-DTPA-enhanced liver magnetic resonance imaging. World J Gastroenterol. (2018) 24:2024–35. doi: 10.3748/wjg.v24.i18.2024 PMC5949715 29760545

[34] Fisher CD Lickteig AJ Augustine LM Oude Elferink RP Besselsen DG Erickson RP. Experimental non-alcoholic fatty liver disease results in decreased hepatic uptake transporter expression and function in rats. Eur J Pharmacol. (2009) 613:119–27. doi: 10.1016/j.ejphar.2009.04.002 PMC2739623 19358839

[35] Lickteig AJ Fisher CD Augustine LM Aleksunes LM Besselsen DG Slitt AL. Efflux transporter expression and acetaminophen metabolite excretion are altered in rodent models of nonalcoholic fatty liver disease. Drug Metab Dispos. (2007) 35:1970–8. doi: 10.1124/dmd.107.015107 17640958

[36] Aierken Y Kong LX Li B Liu XJ Lu S Yang JY. Liver fibrosis is a major risk factor for liver regeneration: A comparison between healthy and fibrotic liver. Med (Baltimore). (2020) 99:e20003. doi: 10.1097/MD.0000000000020003 PMC12245295 32481371

[37] Cordero-Espinoza L Huch M. The balancing act of the liver: tissue regeneration versus fibrosis. J Clin Invest. (2018) 128:85–96. doi: 10.1172/JCI93562 29293095 PMC5749503

[38] Huebert RC Shah VH. Sinusoidal endothelial cells direct traffic at the intersection of regeneration and fibrosis. Hepatology. (2014) 60:754–6. doi: 10.1002/hep.27116 PMC4110160 24615996

[39] Wiemann SU Satyanarayana A Tsahuridu M Tillmann HL Zender L Klempnauer J. Hepatocyte telomere shortening and senescence are general markers of human liver cirrhosis. FASEB J. (2002) 16:935–42. doi: 10.1096/fj.01-0977com 12087054

[40] Hamano M Ezaki H Kiso S Furuta K Egawa M Kizu T. Lipid overloading during liver regeneration causes delayed hepatocyte DNA replication by increasing ER stress in mice with simple hepatic steatosis. J Gastroenterol. (2014) 49:305–16. doi: 10.1007/s00535-013-0780-7 PMC3925298 23512345

[41] Yoon JH Lee JM Kim E Okuaki T Han JK. Quantitative liver function analysis: volumetric T1 mapping with fast multisection B(1) inhomogeneity correction in hepatocyte-specific contrast-enhanced liver MR imaging. Radiology. (2017) 282:408–17. doi: 10.1148/radiol.2016152800 27697007

[42] Gruttadauria S Parikh V Pagano D Tuzzolino F Cintorino D Miraglia R. Early regeneration of the remnant liver volume after right hepatectomy for living donation: a multiple regression analysis. Liver Transpl. (2012) 18:907–13. doi: 10.1002/lt.23450 22505370

[43] Kim JE Kim JH Park SJ Choi SY Yi NJ Han JK. Prediction of liver remnant regeneration after living donor liver transplantation using preoperative CT texture analysis. Abdom Radiol (NY). (2019) 44:1785–94. doi: 10.1007/s00261-018-01892-2 30612157

[44] Meier M Andersen KJ Knudsen AR Nyengaard JR Hamilton-Dutoit S Mortensen FV. Liver regeneration is dependent on the extent of hepatectomy. J Surg Res. (2016) 205:76–84. doi: 10.1016/j.jss.2016.06.020 27621002

[45] Zhang T Wei Y He X Yuan Y Yuan F Ye Z. Prediction of remnant liver regeneration after right hepatectomy in patients with hepatocellular carcinoma using preoperative CT texture analysis and clinical features. Contrast Media Mol Imaging. (2021) 2021:5572470. doi: 10.1155/2021/5572470 34220379 PMC8213498

[46] Ma HY Dong L Quan SZ Li RY Wang XR. Comparison of four markers of hepatic fibrosis and hepatic function indices in patients with liver cirrhosis and hepatoma. Ann Palliat Med. (2021) 10:4108–21. doi: 10.21037/apm-20-1623 33832299

[47] Matsumoto S Okuda S Yamada Y Suzuki T Tanimoto A Nozaki A. Myocardial T1 values in healthy volunteers measured with saturation method using adaptive recovery times for T1 mapping (SMART1Map) at 1.5 T and 3 T. Heart Vessels. (2019) 34:1889–94. doi: 10.1007/s00380-019-01401-5 30976924

[48] Slavin GS Stainsby JA. True T1 mapping with SMART1Map (saturation method using adaptive recovery times for cardiac T1 mapping): a comparison with MOLLI. J Cardiovasc Magnet Resonance. (2013) 15:P3. doi: 10.1186/1532-429X-15-S1-P3

[49] Keenan BP Fong L Kelley RK. Immunotherapy in hepatocellular carcinoma: the complex interface between inflammation, fibrosis, and the immune response. J Immunother Cancer. (2019) 7:267. doi: 10.1186/s40425-019-0749-z 31627733 PMC6798343

[50] Zhu AX Finn RS Edeline J Cattan S Ogasawara S Palmer D. Pembrolizumab in patients with advanced hepatocellular carcinoma previously treated with sorafenib (KEYNOTE-224): a non-randomised, open-label phase 2 trial. Lancet Oncol. (2018) 19:940–52. doi: 10.1016/S1470-2045(18)30351-6 29875066

[51] Yokoi H Isaji S Yamagiwa K Tabata M Sakurai H Usui M. Donor outcome and liver regeneration after right-lobe graft donation. Transpl Int. (2005) 18:915–22. doi: 10.1111/j.1432-2277.2005.00158.x 16008740

[52] Gong WF Zhong JH Lu Z Zhang QM Zhang ZY Chen CZ. Evaluation of liver regeneration and post-hepatectomy liver failure after hemihepatectomy in patients with hepatocellular carcinoma. Biosci Rep. (2019) 39. doi: 10.1042/BSR20190088 PMC6706596 31383787

[53] Haga J Shimazu M Wakabayashi G Tanabe M Kawachi S Fuchimoto Y. Liver regeneration in donors and adult recipients after living donor liver transplantation. Liver Transpl. (2008) 14:1718–24. doi: 10.1002/lt.21622 19025926

[54] Zappa M Dondero F Sibert A Vullierme MP Belghiti J Vilgrain V. Liver regeneration at day 7 after right hepatectomy: global and segmental volumetric analysis by using CT. Radiology. (2009) 252:426–32. doi: 10.1148/radiol.2522080922 19703882

[55] de Graaf W Bennink RJ Heger M Maas A de Bruin K van Gulik TM. Quantitative assessment of hepatic function during liver regeneration in a standardized rat model. J Nucl Med. (2011) 52:294–302. doi: 10.2967/jnumed.110.078360 21233172

[56] Li Q Zhang T Che F Yao S Gao F Nie L. Intravoxel incoherent motion diffusion weighted imaging for preoperative evaluation of liver regeneration after hepatectomy in hepatocellular carcinoma. Eur Radiol. (2023) 33:5222–35. doi: 10.1007/s00330-023-09496-1 36892648

