# Supplementary material for: Preoperative assessment of liver regeneration using T1 mapping and the functional liver imaging score derived from Gd-EOB-DTPA-enhanced magnetic resonance for patient with hepatocellular carcinoma after hepatectomy
Source: Front Immunol. 2025 Jan 30;16:1516848. doi: 10.3389/fimmu.2025.1516848 (PMC11821634; doi:10.3389/fimmu.2025.1516848)
Supplement: Supplementary file 1 [file DataSheet1.docx]

**Table S1 the values of T1-pre (ms), T1-HBP(ms) and Δ% of all the 60 patients included in the study**

| Patients | T1_pre_ (ms) | T1_HBP_(ms) | ∆% |
| --- | --- | --- | --- |
| 1 | 1174.277 | 627.2 | 46.59 |
| 2 | 1060.347 | 457.9167 | 56.81 |
| 3 | 980.9767 | 443.7833 | 54.76 |
| 4 | 1193.75 | 613.88 | 48.58 |
| 5 | 988.81 | 584.0267 | 40.94 |
| 6 | 885.08 | 421.8067 | 52.34 |
| 7 | 958.7233 | 556.4633 | 41.96 |
| 8 | 1107.273 | 573.56 | 48.2 |
| 9 | 927.9433 | 402.0133 | 56.68 |
| 10 | 905.62 | 378.9967 | 58.15 |
| 11 | 1030.377 | 480.8267 | 53.33 |
| 12 | 1015.153 | 549.5267 | 45.87 |
| 13 | 1054.74 | 538.61 | 48.93 |
| 14 | 898.95 | 353.24 | 60.71 |
| 15 | 923.1633 | 429.7433 | 53.45 |
| 16 | 924.57 | 498.7367 | 46.06 |
| 17 | 1064.28 | 444.71 | 58.21 |
| 18 | 969.18 | 489.9 | 49.45 |
| 19 | 971.0333 | 417.2367 | 57.03 |
| 20 | 1029.42 | 480.63 | 53.31 |
| 21 | 921.7867 | 371.1667 | 59.73 |
| 22 | 919.0433 | 482.2033 | 47.53 |
| 23 | 1008.547 | 421.72 | 58.19 |
| 24 | 906.8633 | 420.0833 | 53.68 |
| 25 | 1030.7 | 458.36 | 55.53 |
| 26 | 935.9733 | 490.0667 | 47.64 |
| 27 | 952.83 | 528.4067 | 44.54 |
| 28 | 974.6467 | 506.0133 | 48.08 |
| 29 | 900.1 | 399.25 | 55.64 |
| 30 | 1046.42 | 515.485 | 50.74 |
| 31 | 1005.72 | 523.15 | 47.98 |
| 32 | 964.5533 | 562.2867 | 41.7 |
| 33 | 1085.37 | 468 | 56.88 |
| 34 | 1027.55 | 422.615 | 58.87 |
| 35 | 927.2467 | 489.8833 | 47.17 |
| 36 | 1049.987 | 448.2233 | 57.31 |
| 37 | 983.5433 | 419.5433 | 57.34 |
| 38 | 956.6933 | 551.4233 | 42.36 |
| 39 | 1017.777 | 558.5633 | 45.12 |
| 40 | 1059.523 | 530.13 | 49.97 |
| 41 | 902.5433 | 486.8833 | 46.05 |
| 42 | 1017.373 | 512.89 | 49.59 |
| 43 | 969.13 | 578.48 | 40.31 |
| 44 | 1054.087 | 548.015 | 48.01 |
| 45 | 1003.235 | 469.47 | 53.2 |
| 46 | 1082.373 | 569.74 | 47.36 |
| 47 | 946.4 | 433.71 | 54.17 |
| 48 | 937.6467 | 424.38 | 54.74 |
| 49 | 836.2667 | 490.47 | 41.35 |
| 50 | 834.22 | 357.0167 | 57.2 |
| 51 | 984.54 | 546.02 | 44.54 |
| 52 | 971.08 | 365.24 | 62.39 |
| 53 | 934.8767 | 542.84 | 41.93 |
| 54 | 869.5467 | 434.5 | 50.03 |
| 55 | 1048.32 | 503.3433 | 51.99 |
| 56 | 1040.895 | 454.53 | 56.33 |
| 57 | 1042.123 | 548.61 | 47.36 |
| 58 | 1092.925 | 521.175 | 52.31 |
| 59 | 1140.073 | 506.2 | 55.6 |
| 60 | 1112.84 | 537.37 | 51.71 |

**Correlations Between T1 mapping Parameters, Inflammation grade and Liver Regeneration**

Whether in the total patients or subgroups undergoing minor or major hepatectomy, all T1 mapping parameters did not show significant correlations with inflammation grade (all *P*>0.05, Table S2). Meanwhile, no statistically significant correlation was observed between inflammation grade and RI (all *P*>0.05, Table S2).

**Table S1. Results of Correlations Between the T1 mapping parameters and inflammation grade, RI and inflammation grade**

| **Variables** | **Total patients**  **(n=60)** | |  |  | **Minor hepatectomy**  **(n=35)** | |  | | |  | | **Major hepatectomy**  **(n=25)** | | |
| --- | --- | --- | --- | --- | --- | --- | --- | --- | --- | --- | --- | --- | --- | --- |
|  | **Correlation coefficient** | ***P* value** |  |  | **Correlation coefficient** | ***P* value** |  |  |  | | **Correlation coefficient** | | | ***P* value** |
| T1pre (ms) | 0.157 | 0.157 |  |  | 0.018 | 0.897 |  | | |  | | 0.262 | 0.108 | |
| T1_HBP_(ms) | 0.200 | 0.053 |  |  | 0.233 | 0.091 |  | | |  | | 0.169 | 0.300 | |
| ∆% | -0.139 | 0.178 |  |  | -0.174 | 0.205 |  | | |  | | -0.059 | 0.717 | |
| FLIS | -0.155 | 0.173 |  |  | -0.293 | 0.053 |  | | |  | | -0.005 | 0.978 | |
| RI (%) | -0.116 | 0.261 |  |  | -0.011 | 0.952 |  | | |  | | -0.174 | 0.288 | |

**Note**: T1-pre, T1 relaxation time of the liver before gadoxetic acid injection; T1-HBP, T1 relaxation time of the liver 20 min after gadoxetic acid injection; ∆%, the reduction rate of T1 relaxation time; FLIS: a functional liver imaging score;RI, regeneration index

**Figure S1. The relation between T1 mapping parameters and steatosis grade (A), FLIS and steatosis grade (B), steatosis grade and RI (C) in total patients.**

(RI, regeneration index; T1_pre_, T1 relaxation time of the liver before gadoxetic acid injection; T1_HBP_, T1 relaxation time of the liver 20 min after gadoxetic acid injection; ∆%, the reduction rate of T1 relaxation time; FLIS a functional liver imaging score)


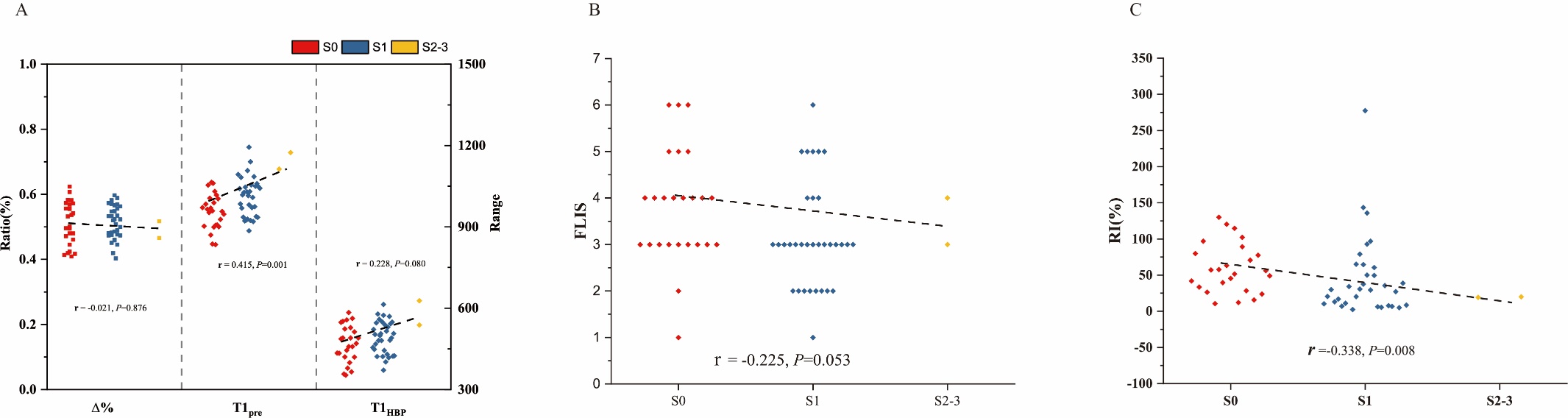


**Figure S2. The relation between T1 mapping parameters and steatosis grade (A), FLIS and steatosis grade (B), steatosis grade and RI (C) in patients undergoing minor hepatectomy.**

(RI, regeneration index; T1_pre_, T1 relaxation time of the liver before gadoxetic acid injection; T1_HBP_, T1 relaxation time of the liver 20 min after gadoxetic acid injection; ∆%, the reduction rate of T1 relaxation time; FLIS a functional liver imaging score)


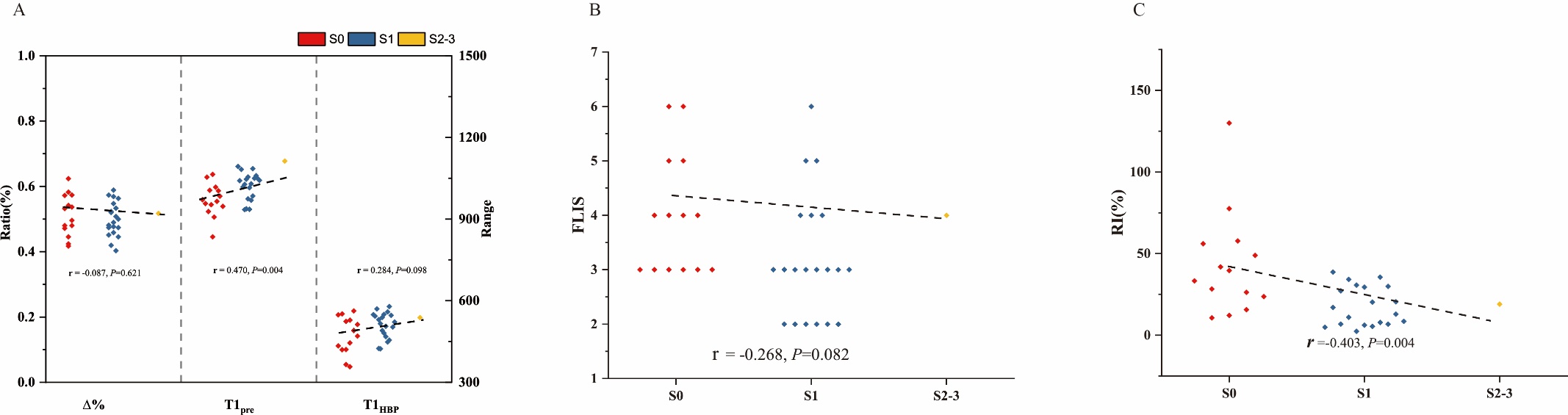


**Figure S3. The relation between T1 mapping parameters and steatosis grade (A), FLIS and steatosis grade (B), steatosis grade and RI (C) in patients undergoing major hepatectomy.**

(RI, regeneration index; T1_pre_, T1 relaxation time of the liver before gadoxetic acid injection; T1_HBP_, T1 relaxation time of the liver 20 min after gadoxetic acid injection; ∆%, the reduction rate of T1 relaxation time; FLIS a functional liver imaging score)


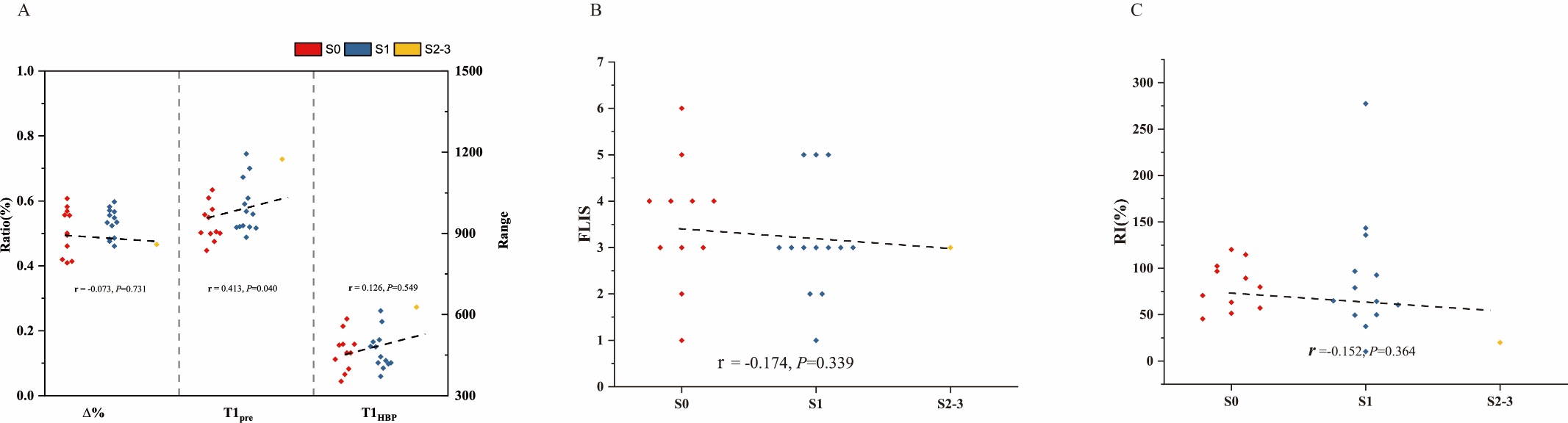


**Figure S4. The relation between PHRR and LV_pre_(A), PHRR and the type of hepatectomy (B), LV_pre_ and the type of hepatectomy(C) in the total patients**

(PHRR, parenchymal hepatic resection rate; LV_pre_, volume of future remnant liver)


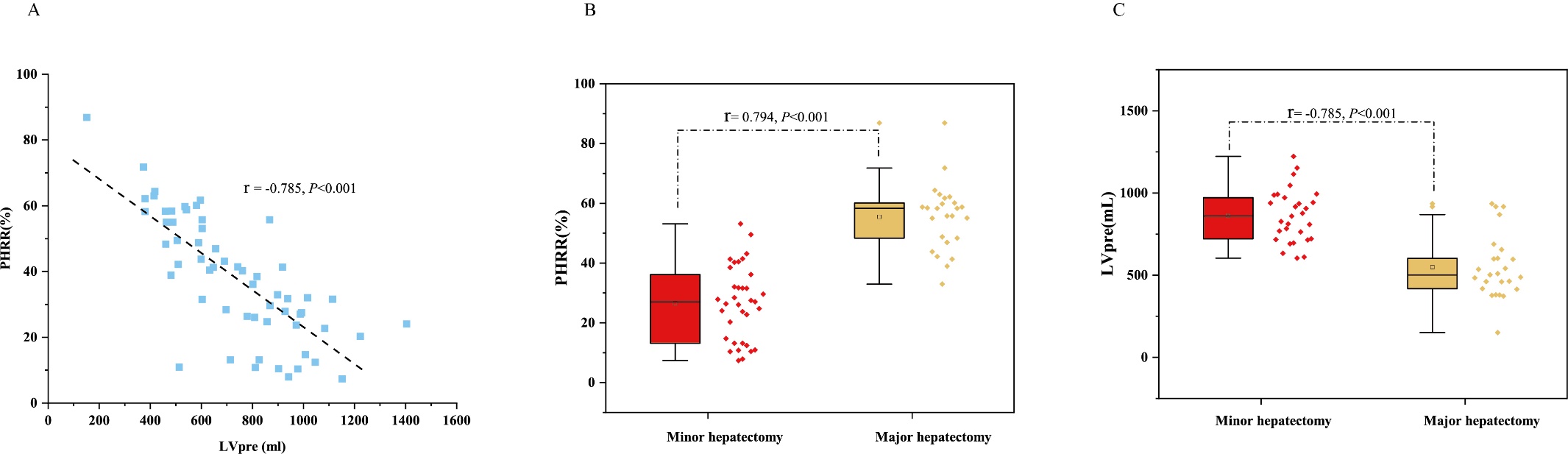


**Figure S5. The relation between T1_pre_ and T1_HBP_(A), T1_pre_ and ∆%(B), T1_HBP_ and ∆%(C), FLIS and T1_pre_(D)_,_ FLIS and T1_HBP_(E)_,_ FLIS and ∆%(F) in the total patients**


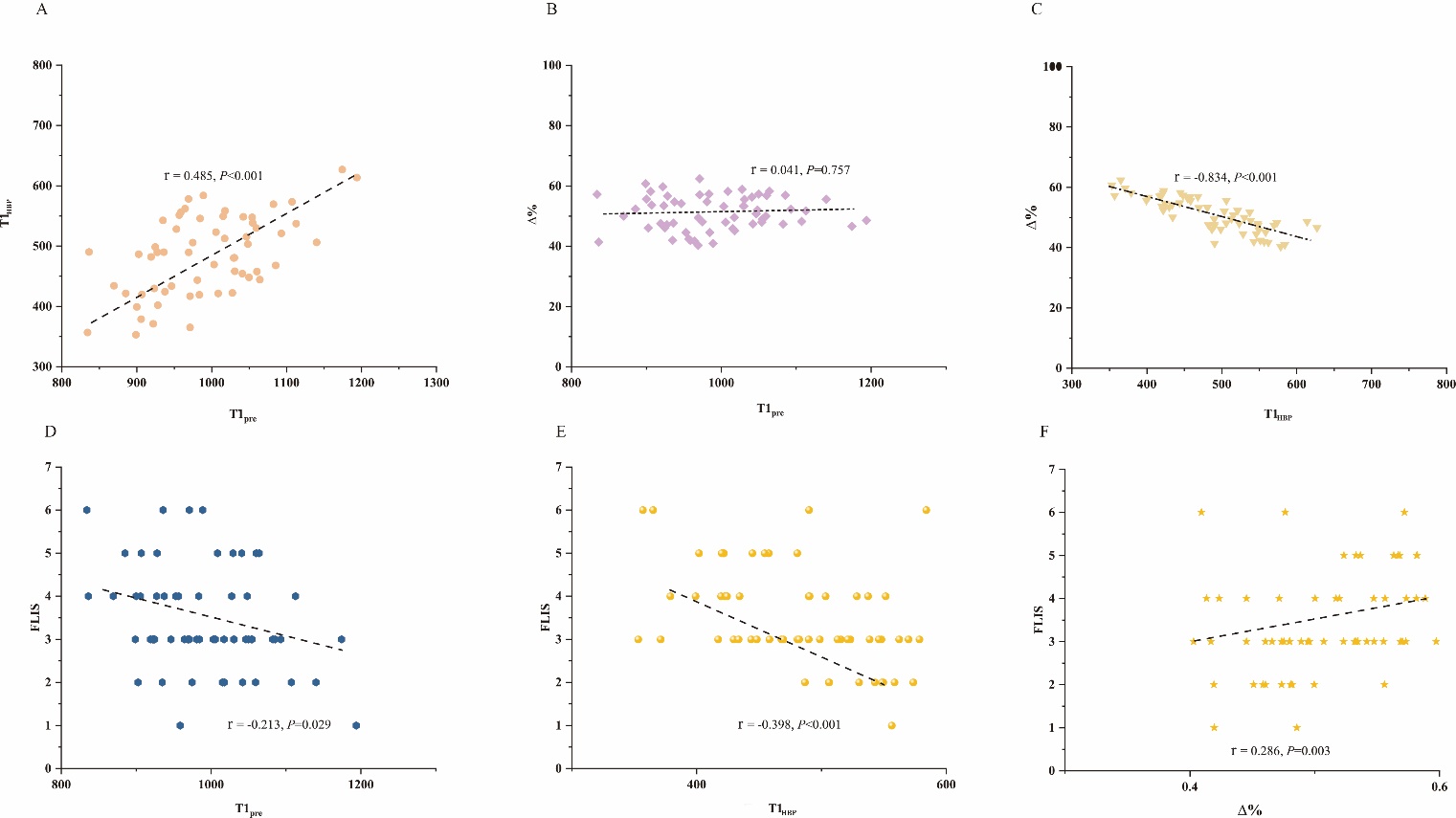
(T1_pre_, T1 relaxation time of the liver before gadoxetic acid injection; T1_HBP_, T1 relaxation time of the liver 20 min after gadoxetic acid injection; ∆%, the reduction rate of T1 relaxation time; FLIS a functional liver imaging score)

**Figure S6. The relation between PHRR and LV_pre_(A),T1_pre_ and T1_HBP_(B), T1_pre_ and ∆%(C), T1_HBP_ and ∆%(D), FLIS and T1_pre_(E)_,_ FLIS and T1_HBP_(F)_,_ FLIS and ∆%(G) in patients undergoing minor hepatectomy**

(PHRR, parenchymal hepatic resection rate; LV_pre_, volume of future remnant liver;T1_pre_, T1 relaxation time of the liver before gadoxetic acid injection; T1_HBP_, T1 relaxation time of the liver 20 min after gadoxetic acid injection; ∆%, the reduction rate of T1 relaxation time; FLIS a functional liver imaging score)


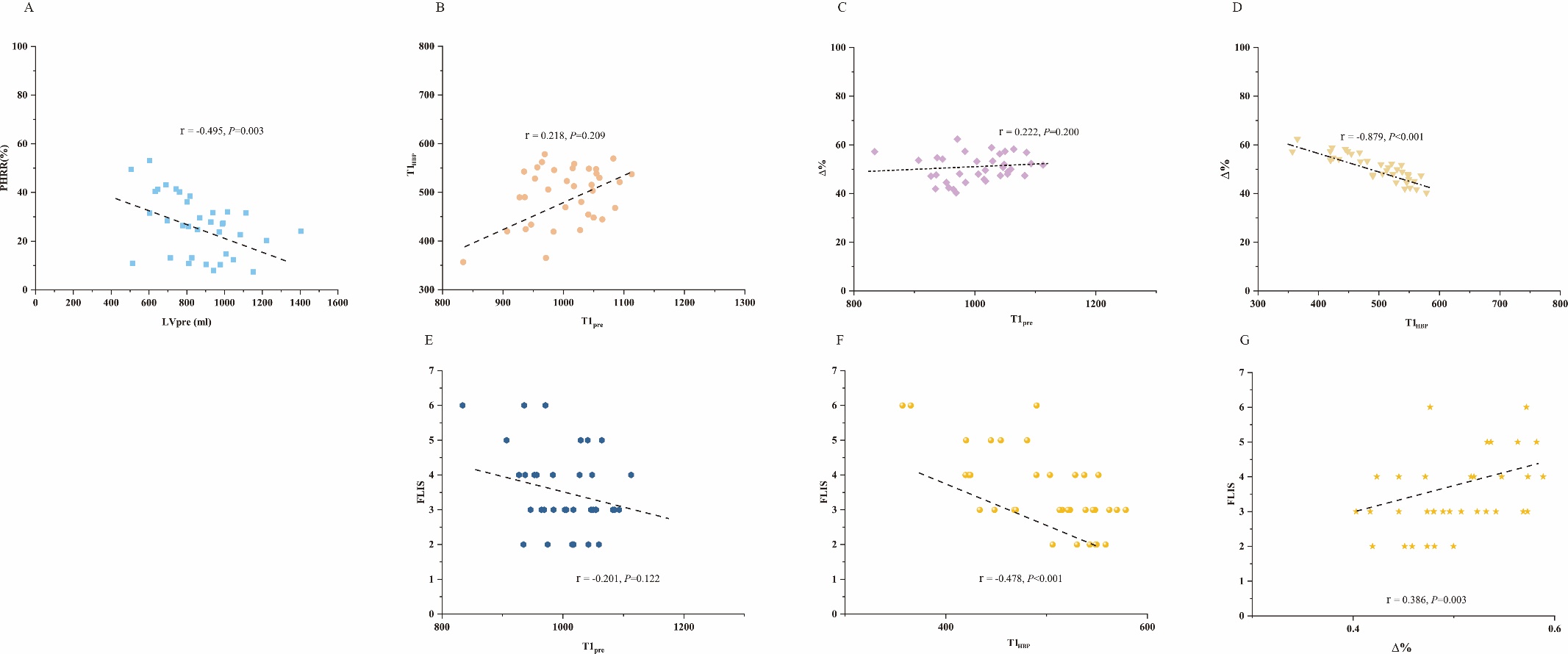


**Figure S7. The relation between PHRR and LV_pre_(A),T1-pre and T1_HBP_(B), T1_pre_ and ∆%(C), T1_HBP_ and ∆%(D), FLIS and T1_pre_(E)_,_ FLIS and T1_HBP_(F)_,_ FLIS and ∆%(G) in patients undergoing major hepatectomy.**


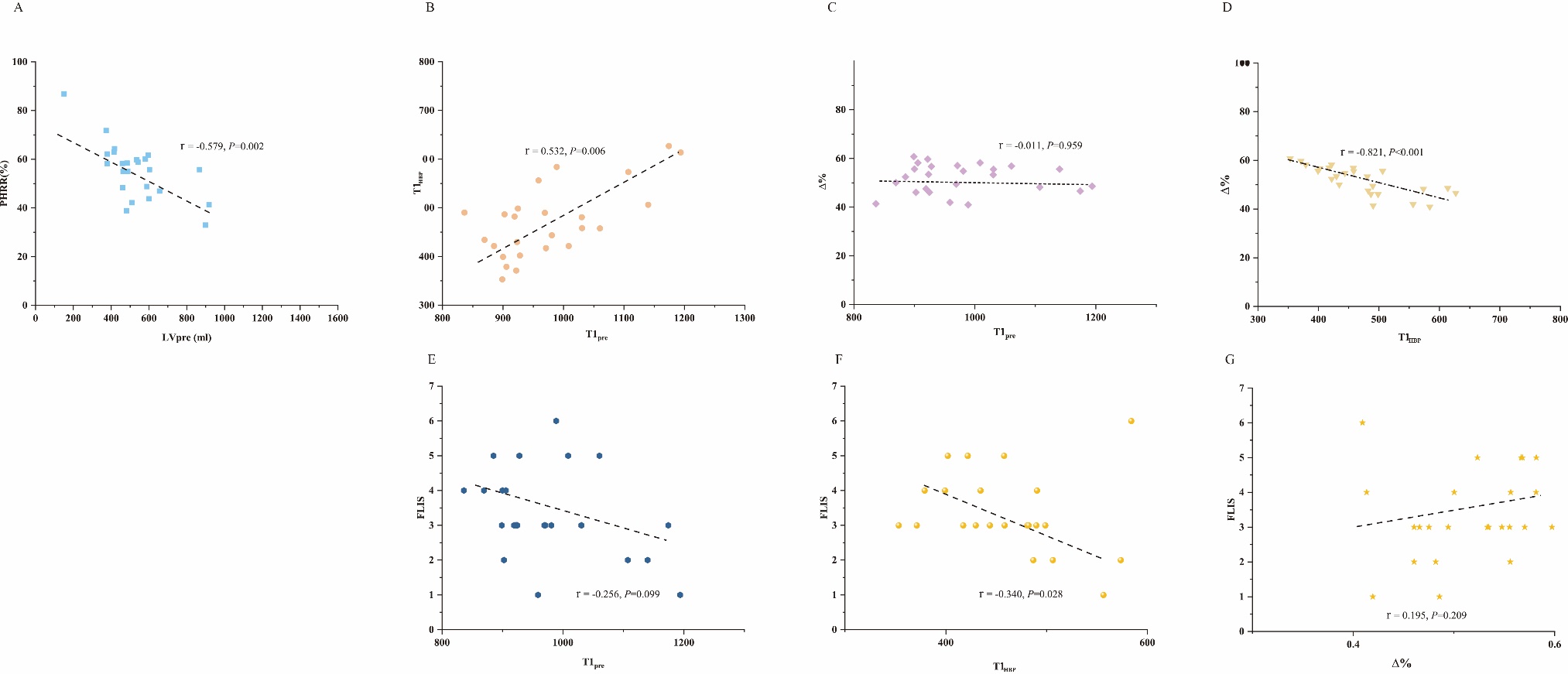
(PHRR, parenchymal hepatic resection rate; LV_pre_, volume of future remnant liver;T1_pre_, T1 relaxation time of the liver before gadoxetic acid injection; T1_HBP_, T1 relaxation time of the liver 20 min after gadoxetic acid injection; ∆%, the reduction rate of T1 relaxation time; FLIS a functional liver imaging score)
